# Inequalities in Older age and Primary Health Care Utilization in Low- and Middle-Income Countries: A Systematic Review

**DOI:** 10.1177/00207314211041234

**Published:** 2021-10-21

**Authors:** Qian Gao, A. Matthew Prina, Yuteng Ma, David Aceituno, Rosie Mayston

**Affiliations:** 1 4616 King’s College London, London, UK; 2 4919 University College London, London, UK

**Keywords:** health care utilization, primary health care, socioeconomic status, low- and middle- income countries, aging

## Abstract

The objective of this research was to systematically review and synthesize quantitative studies that assessed the association between socioeconomic inequalities and primary health care (PHC) utilization among older people living in low- and middle- income countries (LMICs). Six databases were searched, including Embase, Medline, Psych Info, Global Health, Latin American and Caribbean Health Sciences Literature (LILACS), and China National Knowledge Infrastructure, CNKI, to identify eligible studies. A narrative synthesis approach was used for evidence synthesis. A total of 20 eligible cross-sectional studies were included in this systematic review. The indicators of socioeconomic status (SES) identified included income level, education, employment/occupation, and health insurance. Most studies reported that higher income, higher educational levels and enrollment in health insurance plans were associated with increased PHC utilization. Several studies suggested that people who were unemployed and economically inactive in older age or who had worked in formal sectors were more likely to use PHC. Our findings suggest a pro-rich phenomenon of PHC utilization in older people living in LMICs, with results varying by indicators of SES and study settings.

The Sustainable Development Goals (SDGs) and Alma Ata Declaration recommend health for all, regardless of economic status, age, or other characteristics.^1^ Older people are a vulnerable population group who are more likely to be impoverished,^2,3^ including in developing countries.^4^ Globally, governments are working toward universal health coverage (UHC), with achievements made in increasing the coverage of essential health services by ≈20% from 2000 to 2015. However, half of the world's population still lack full coverage,^5^ and wealthy people continue to have better access to health care^6^ and better health outcomes.^7,8^ For example, in China, the gap in health service utilization between rich and poor is documented in increased use of both outpatient care and inpatient health services by wealthier people.^6^ Many global health targets focus on younger age groups.^9,10^ Therefore, there is a danger that older people, particularly those who are poor, may be left behind by health goals and reforms. Under this context, primary health care (PHC) plays a vital role in bridging the gap for achieving “health for all.” The concept of PHC that was proposed in the Declaration of Alma-Ata has been widely cited in different contexts^11^ as a fundamental component of an equality orientated and sustainable health system. The World Health Organization (WHO) defined it as “a whole-of-society approach to health that aims to ensure the highest possible level of health and well-being and their equitable distribution by focusing on people's needs and preferences (as individuals, families, and communities) as early as possible along the continuum from health promotion and disease prevention to treatment, rehabilitation and palliative care, and as close as feasible to people's everyday environment”.^12^

Older people require care that is integrated, local, and well-aligned to needs that arise from problems common to older age: multimorbidity, declines in mobility, and other impairments.^13,14^ These needs are challenging for governments and families to address as primary care is principally designed to meet goals related to maternal and child health and infectious disease.^12^ As populations age rapidly all over the world, the proportion of people aged 60 years and older is expected to increase from 12% in 2015 to 22% in 2050.^13^ Alongside the demographic transition, epidemiological transitions mean that noncommunicable diseases are becoming more common, with co-morbidities increasing progressively with age.^15,16^ At the same time, for many low- and middle-income countries’ (LMICs’) health systems, infectious disease—particularly chronic infectious diseases, such as human immunodeficiency virus and tuberculosis—remain prevalent.^17,18^ This shift toward an increasing burden of chronic disease requires robust PHC in communities,^19^ with chronic care models to meet population health care needs.^20^ However, many health systems now have a double burden in dealing with both infectious disease and noncommunicable diseases, with health systems less well-equipped to address the management of chronic illness^21^ and therefore failing to address the health care needs of older people.^22,23^

Socioeconomic inequalities are differences in income, social class, and occupational and educational background^24^ associated with disparities, where those with more disadvantaged backgrounds are more likely to experience adverse outcomes such as premature mortality, multiple chronic illnesses, and disability.^25^ Inequalities are pervasive and resistant to government intervention. For example, evidence from Ghana has shown that wealth inequalities remain in older people's health services utilization after implementation of the national health insurance plan, with the poorest older people benefitting the least from this policy shift.^26^ Socioeconomic inequalities accumulate over the life course to negatively influence health outcomes in later life.^25,27,28^ However, there is evidence that high-quality PHC offers opportunities to mitigate the effects of socioeconomic inequalities.^29^ In particular, PHC is fundamental to responding to the needs of older people, as it is best placed to deliver effective care in community settings.^12^ A solid and robust PHC system enables care integration and coordination for older populations and supports collaboration across sectors and between different levels of the health care system,^30^ both of which are essential for the effective management of multimorbid chronic conditions. As a socioeconomically disadvantaged group, older people experience more barriers in accessing health services; socioeconomic inequalities such as low income and a lack of health insurance are driving factors in restricting older people's health care use.^31^ Access to PHC would seem to be a key determinant for achieving the SDGs and UHC.^32^ Therefore, it is essential to improve the equity of PHC for older people regardless of their socioeconomic position.

Older people with low socioeconomic status (SES) tend to be at risk of not accessing health care and having unmet health needs, especially those living in health resource-limited settings. A few systematic reviews have been conducted to synthesize evidence about socioeconomic differences in health services utilization, but most do not focus on the older age group and/or are global, with insufficient focus on LMICs. For example, an earlier systematic review from Europe highlighted socioeconomic inequalities and health care access in Central and Eastern Europe and in the Commonwealth of Independent States, but this was not limited to older populations.^33^ A more recent review focused on older adults’ utilization of health services, but because it was global and included all health services, little detail was provided on LMICs and primary care utilization.^34^ Overall, there is still limited evidence about the equity of primary health care utilization among older people, especially in LMICs. It remains to be seen how socioeconomic inequality affects older people's PHC utilization. In this review, we included non-traditional databases (eg, the China National Knowledge Infrastructure [CNKI] and Latin American and Caribbean Health Sciences Literature [LILACS] databases), to better capture publications in other languages from China and Latin America, regions which are now major contributors to the evidence base in this area but often neglected from search strategies. Aligning with the UHC and SDG-3 goal, this systematic review aims to synthesize the available quantitative evidence on the relationship between socioeconomic inequalities and PHC utilization among older people (60 years old or above) living in LMICs.

## Materials and Methods

### Search Strategy

The systematic review was registered on PROSPERO (registration number: CRD420191 19969). The Preferred Reporting Items for a Systematic Review and Meta-Analysis (PRISMA) guidelines were followed (S1 Appendix). Six databases were manually searched, including English databases (ie, Embase, Medline, Psych Info, Global Health); the Virtual Health Library, for searching the LILACS database to identify relevant research in Portuguese and Spanish; and the CNKI database, to identify Chinese literature. Searching strategies for these databases were translated and adapted. To ensure that the search was as comprehensive as possible, each database was searched using both Medical Subject Headings (MeSH terms) and synonyms. Articles published before January 2019 were considered for inclusion. Search terms were used across a range of relevant databases and adapted for each database used. The search strategy involved combining search terms for: “socioeconomic status (SES)” AND “primary health care utilization” AND “older people” AND “low- and middle-income countries (LMICs).” Additional articles were identified by backward citation tracking for each relevant retrieved article. The searching strategy is detailed in S2 Appendix.

### Inclusion and Exclusion Criteria

Eligible articles included those describing quantitative studies with participants aged 60 years and above, carried out in LMICs, as defined by the World Bank during the year they were conducted. We focused on quantitative studies where both indicators of SES and PHC utilization were measured and reported. We considered socioeconomic exposures, including established SES indicators (education, income, and employment/occupation), insurance status/government financial support, and other economic domains (ie, social class, poverty, income inequality, deprivation, and assets index). PHC refers to the services delivered in first-level health platforms. We defined PHC according to the WHO conceptual framework of PHC^12^ and took account of all types of community-located services in this review, including health service/care delivered in PHC platforms (community-based care center, health center/station, and first-level hospital) and health services provided by non-specialist primary care workers, general practitioners (GPs), and traditional healers. Public health programs, population-based interventions, and community-based development programs (health-related and those covered by the national health service system) were also considered in this review. There were no language or time restrictions in our searching procedures. For studies from the same cohort that captured the same population but in different years, we included the paper with the larger sample. For studies carried out in the general population, only those where it was possible to extract data for the older age group (60 years and above) were included. All articles identified by database searches were screened by two reviewers according to the inclusion and exclusion criteria (details in Table S1).

### Data Extraction and Quality Assessment

Results from the database search were exported to Rayyan (http://rayyan.qcri.org).^35^ The screening was carried out using title and abstract screening followed by full-text screening. After the full-text screening, a final list of selected articles was imported to Endnote. The screening was carried out independently by two reviewers (QG and YM; QG and DA) to identify whether studies met inclusion criteria. Following that, all eligible studies in English and Chinese were extracted by the lead reviewer (QG), while eligible papers in Portuguese and Spanish were extracted by a second reviewer (DA). Extracted information included author name(s); year of publication; language; region; study setting; objectives of the study; study population; study design; sample information (sample size, participants’ age, and setting); recruitment and study completion rates; original studies outcome; studied outcome (PHC utilization); outcome measure; exposure (indicators of SES); exposure measure; evaluated confounders and statistical information (ie, crude effect size; adjusted effect size, and 95% confidence intervals). Results of statistical significance tests were reported if odds ratios were not reported. Two reviewers assessed and scored the quality of all eligible papers using the Joanna Briggs Institute (JBI) critical appraisal tool. JBI's critical appraisal checklist for cross-sectional studies assesses eight potential domains of bias, including inclusion criteria, study subjects and setting, exposure measurement, condition measurement, confounder measurement, strategies to deal with confounder, outcome measurement, and statistical analysis.^36^ We assessed the quality of eligible studies into three categories: low quality, moderate quality, and high quality, according to JBI criteria related to the above eight domains. Any disagreements in screening and quality ratings by two reviewers were resolved by discussion and consensus with research group leaders (RM and MP).

### Data Synthesis

A narrative synthesis was carried out by grouping and analyzing results (any types of PHC services utilization) by different categories of SES indicators (individual and household-level income, education, current employment status/occupation, and health insurance). To understand the context of health care systems in the studied countries and to estimate their progress toward UHC, we extracted the UHC global monitoring data from WHO and the World Bank 2017 monitoring report^5^ and included the UHC essential services coverage index (an indicator for monitoring SDG 3.8.1) in our synthesis. The index ranged from 0 to 100, with higher index indicating higher coverage rate.^5^

## Results

### Study Characteristics

**Figure 1.**
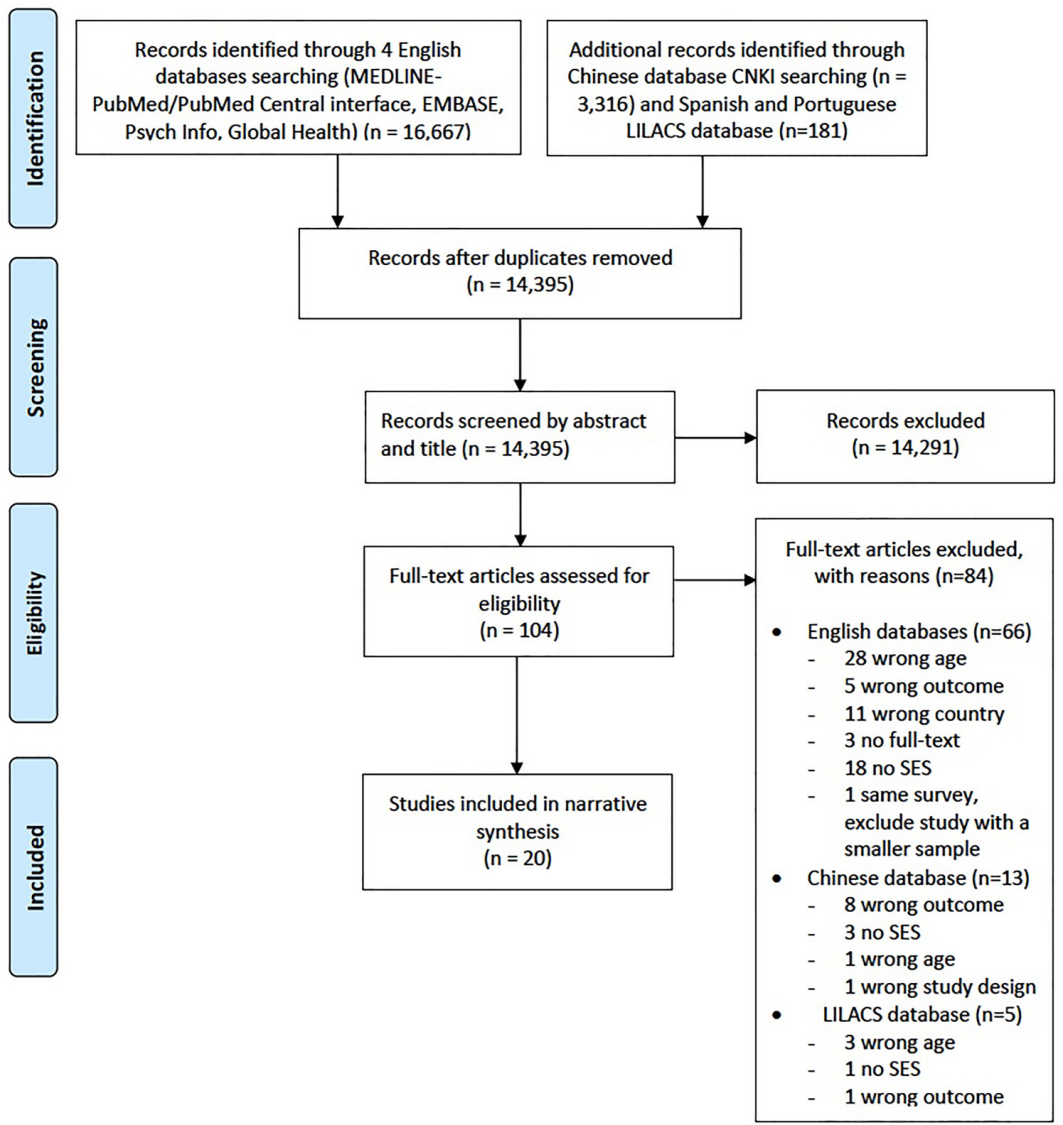
Preferred Reporting Items for a Systematic Review and Meta-Analysis Flow Diagram.

A total of 20 164 articles were indexed initially. After removing 5769 duplicates, we reviewed 14 395 titles and abstracts and screened 104 full texts. Finally, 20 articles were found to be eligible for inclusion (The PRISMA Flow Diagram is shown in Figure 1). All the included articles were cross-sectional studies; a summary of study characteristics is shown in Table 1. Among the 20 eligible studies, 18 studies were published in journals and two were published theses. According to the JBI critical appraisal tool for cross-sectional studies, most studies were of high quality in the following domains: reporting of study subjects, setting and confounder measurement (20 of 20), strategies to deal with confounder (18 of 20), and statistical analysis (16 of 20). Domains with lower quality included inclusion criteria (14 of 20 studies with high quality), exposure and condition measurement (13 of 20 studies with high quality), and outcomes measurement, where most studies were of moderate or low quality (Table S2).

**Table 1.** Characteristics of Included Studies (N = 20).

**Authors (Year)**	**Language**	**Country, Region**	**Original participants’ age (Range & Mean)**	**Study population**	**Sampling method & setting**	**Response rate**	**SES (Types)**	**PHC utilization (Measures)**	**Payment method**
**Alkhawaldeh et al, 2014**	English	Irbid, Jordan	Older adults (aged 50 and older) 64.6 years old (SD = 9.7)	50 + years old (Mean age over 60 years old) (N = 190)	A proportional convenience sampling The catchment areas associated with three comprehensive PHC centers	Not reported	Employment status, educational level, enabling factors included monthly income and health insurance coverage	Use of primary health care in the past 1, 6, and 12 months	Out-of-pocket & health insurance
**Albanese et al, 2011**	English	Urban and rural sites in China, India, Mexico, and Peru; urban sites in Cuba, Dominican Republic, Puerto Rico, and Venezuela; and a rural site in Nigeria	Older adults (65 + years old) Cuba (75.1 years, SD = 7.0); Dominican Republic (75.3 years, SD = 7.5); Puerto Rico (76.3 years, SD = 7.4); Venezuela (72.3 years, SD = 6.9); Peru urban (75.0 years, SD = 7.4); Peru rural (74.2 years, SD = 7.3); Mexico urban (74.5 years, SD = 6.6); Mexico rural (74.1 years, SD = 6.7); China urban (73.9 years, SD = 6.2); China rural (72.4 years, SD = 6.0); India urban (71.3 years, SD = 6.1); China rural (72.6 years, SD = 5.8); Nigeria (72.7 years, SD = 7.6)	65 + years old (N = 17 944)	Systematic sampling procedure Households	Over 80%	Educational level, wealth, health insurance	Use of any community health care services (primary care doctor, hospital-based doctor, private doctor, traditional healer, and other community services)	Cuba: Free Others: Out-of-pocket & health insurance
**Ayele et al, 2017**	English	Ethiopia	Elderly (≥65 years) patients Mean age: NA	65 + years old (N = 324)	Systematic sampling procedure Outpatient clinics	87.80%	Educational status, average monthly income, employment status	Use of complementary and alternative medicine since diagnosed of chronic noncommunicable disease	Pay items Payment method: NA
**Bos et al, 2007**	English	Brazil	60 to 69 years old Mean age: NA	60 to 69 years old (N = 7920)	NA	Not reported	Education, economic sector, occupation, Individual income (log) and family income per capita (log)	Use of primary health care	Private sectors: Out-of-pocket & health insurance Public sectors: free
**Goeppel et al, 2016**	English	China, Ghana, India, Mexico, the Russian Federation, and South Africa	50 years old and above China 64.2 years (SD = 0.2); Ghana 66.3 years (SD = 0.4); India 62.3 years (SD = 0.3); Mexico 64.8 years (SD = 0.9); Russia 65.2 years (SD = 0.7); South Africa 62.4 years (SD = 0.4)	50 + years old (Mean age over 60 years) (N = 16 631) China N = 6558; Ghana N = 1327; India N = 2623; Mexico N = 1341; Russia N = 2916; South Africa N = 1866	Nationally representative samples (using person-level analysis weights based on selection probabilities in the survey sampling design) Households	Ranged from 52% in Mexico to 93% in China	Health insurance	Access to basic chronic care	Out-of-pocket & health insurance
**Macinko et al, 2018**	English	Brazil	50 years old and above 62.99 years old (95%CI 62.16-63.82)	50 + years old (Mean age over 60 years old) N = 9412	Multistage stratified sampling: Sampling plan combined stratification of primary sampling units (municipalities), census tracts, and households Households	Not reported	Household wealth quintiles	Self-reported number of any general practitioner or non-specialist doctor visits in the past 12 months	Private sectors: Out-of-pocket & health insurance Public sectors: free
**Martinez, 2014**	English	Chile	All age Mean age: NA	65 + years old N = 22 473	Multistage sampling technique Households	Not reported	Income deciles, education, employment status	Primary care services utilization (preventive and acute care visits) in the last 3 months	Private sectors: Out-of-pocket & health insurance Public sectors: free
**Polluste et al, 2009**	English	Estonia	15 to 74 years old Mean age: NA	65 to 74 years old N = 1446	Two-stage systematic sampling The primary sampling units were settlements (cities, towns, urban settlements, and villages)	Not reported	Education, income per family member per month	Use of health services (general practitioner [GP]/dentist)	Out-of-pocket & health insurance
**Rodrigues et al, 2009**	English	The south and northeast regions of Brazil	65 + years old with chronic conditions Mean age: NA	65 + years old with chronic conditions N = 2889	Multiple stage stratified sampling Primary health care units	Not reported	Level of schooling (complete years of study) and family income	Use of medical visits (primary health care unit) in the past 6 months	Out-of-pocket & health insurance
**Somkotra et al, 2013**	English	Thailand	60 + years old Mean age: NA	60 + years old N = 20 353 (Year 2003, N = 8951; Year 2009, N = 11 402)	Two-stage stratified sampling Households	Not reported	Household assets index (household quintiles)	Oral health care utilization in the past 12 months	Public sector: without copayment
**Wang et al, 2012**	Chinese	Urumchi, China	60 + years old 68.96 years old, (SD = 8.08)	60 + years old N = 713	Cluster systematic sampling Community	95.10%	Monthly income	Use of community health services during the last year	Out-of-pocket & health insurance
**He et al, 2013**	Chinese	Foshan, China	60 + years old Mean age: NA	60 + years old N = 1534	Stratified random sampling Community	95.76%	Health insurance	Use of community health services during the past year	Out-of-pocket & health insurance
**He et al, 2012**	Chinese	China	65 + years old Mean age: NA	65 + years old N = 1135	Multistage stratified sampling Households	94.75%	Educational attainment, annual per capita income	Use of basic public health service (health checkup)	Free services
**Sun et al, 2013**	Chinese	Tangshan, China	60 + years old 70 years old (SD = 7)	60 + years old N = 3255	Cluster systematic sampling Community health services (CHS) agencies & Township health centers	99.70%	Health insurance, level of education, employment status and household income	Use of community health services in the past year	Out-of-pocket & health insurance
**Wen et al, 2015**	Chinese	Beijing, China	65 + years old 72.13 years old, (SD = 5.51)	65 + years old N = 943	Two-stages cluster systematic sampling Households	99.26%	Occupation	Use of essential public health services	Free services
**Lu et al, 2015**	Chinese	Guiyang, China	60 + years old 71.77 years old (SD = 8.13)	60 + years old N = 509	Stratified random sampling Community	98.45%	Education, monthly income, health insurance	Use of community health services	Out-of-pocket & health insurance
**Xi et al, 2010**	Chinese	Changsha, China	60 + years old Mean age: NA	60 + years old N = 602	Multistage cluster sampling Community	95.56%	Education, monthly income, health insurance, occupation (before 60 years old)	Use of community health services during the past year	Out-of-pocket & health insurance
**Melguizo-Herrera & Castillo-Ávila, 2012**	Spanish	Cartagena, Colombia	60 years old and above Mean = 69.7 (SD:NA)	60 + years old N = 656	Two-stage stratified sampling Primary sampling units: Cartagena city Secondary sampling units: Random sampling from neighborhoods blocks	Not reported	SES	Primary care (general) services utilization in the past month	Out-of-pocket & health insurance
**Paskulin et al, 2011**	Portuguese	Porto Alegre, Brazil	60 years old and above Mean age: NA	60 + years old N = 292	Two-stage probabilistic sampling Primary sampling units: 16 districts from Porto Alegre Secondary sampling units: random sampling of houses of selected districts	80.20%	Education attention	Primary care (general) services utilization in the past 6 months	Private sectors: Out-of-pocket & health insurance Public sectors: free
**Rodrigues et al, 2008**	Portuguese	Brazil	65 years old and above Mean age: NA	65 + years old N = 4003	Multistage probabilistic sampling 41 over 100,000 inhabitants” councils from Brazil	Not reported	Education, monthly family income	PHC utilization in the past month	Private sectors: Out-of-pocket & health insurance Public sectors: free

* NA: Not available.

**Figure 2.**
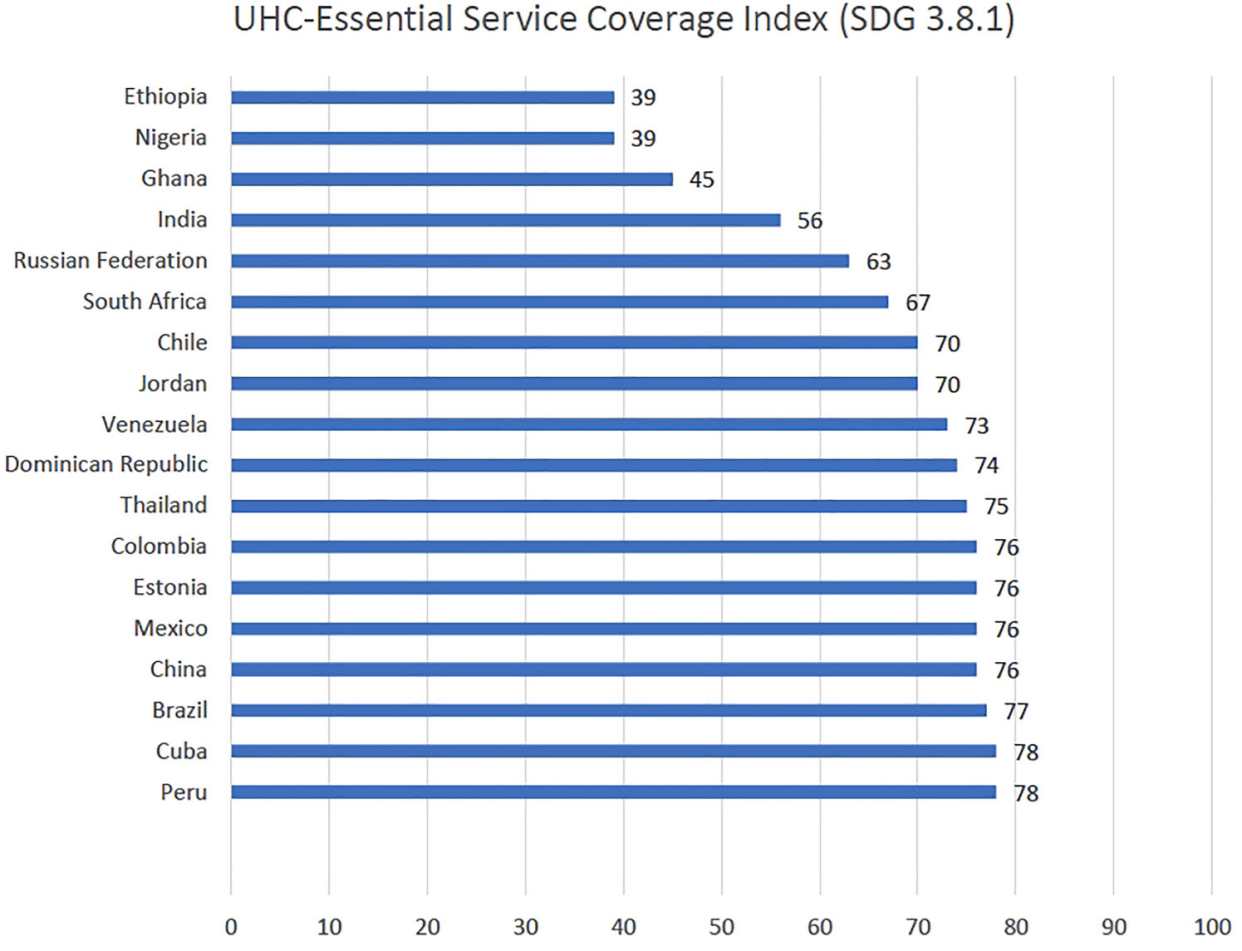
UHC-Essential Service Coverage Index (SDG 3.8.1).

Abbreviations: UHC, universal health coverage; SDG, Sustainable Development Goals.

**Figure 3.**
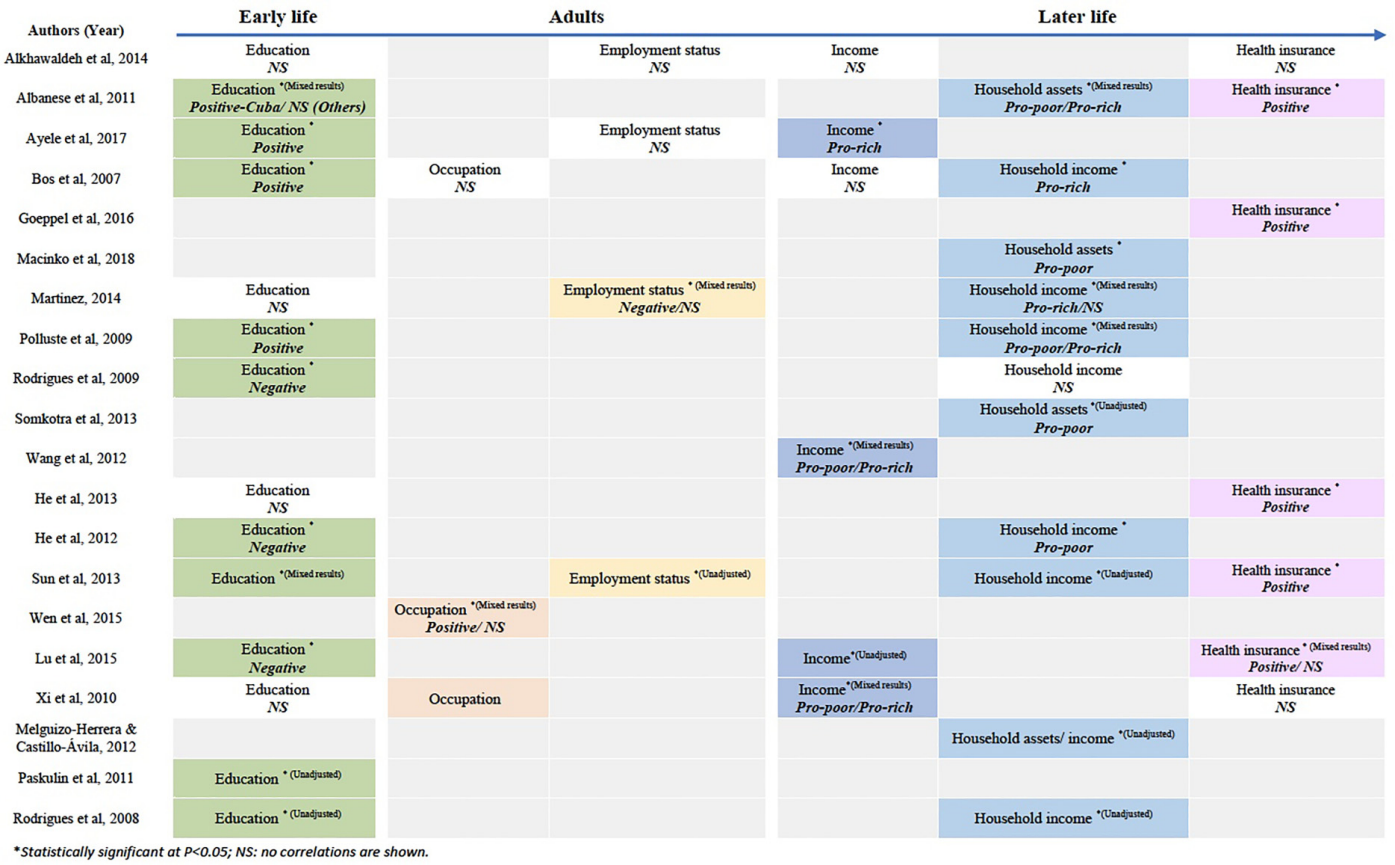
Summary of Associations Found Between SES Indicators and PHC Utilization Among Older Adults in LMICs.

Abbreviations: LMICs, low- and middle-income countries; SES, socioeconomic status; PHC, primary health care.

Of the 20 studies, nearly half were carried out in Asia (N = 9),^37–45^ followed by seven studies conducted in Latin America and the Caribbean.^46–52^ One study was from Europe^53^ and another was from sub-Saharan Africa.^54^ Two studies reported results from multiple countries.^55,56^ Most studies focused on older adults (N = 18, 86.4%), while two studies targeted adults (N = 1) and all ages (N = 1). Four studies captured free PHC services only, from China (essential public health services),^41,43^ Cuba,^55^ and Thailand (PHC in public sectors),^38^ while the other studies included the PHC services from public and/or private sectors under mixed payment methods (out-of-pocket and health insurance coverage) (Table 1). The WHO/World Bank UHC indicators (essential service coverage index, 2015) across studied countries are displayed in Figure 2; the UHC service coverage index was higher (≥75) in countries such as Peru, Cuba, Brazil, China, Mexico, Estonia, Colombia, and Thailand, indicating relatively good progress toward UHC goals in the service coverage dimension.^5^ Measures used for indicators of SES and PHC utilization in each eligible study are described in Table S3. Ten studies were published in English, seven studies were in Chinese, two studies were in Portuguese, and one study was published in Spanish. Studies were conducted between 2008 and 2018. For measuring socioeconomic inequalities among older adults, the most common domains reported were income (N = 16), education (N = 14), employment/occupation (N = 7), and health insurance (N = 7). The majority of studies measured multiple indicators of SES (N = 11). PHC utilization was measured over different time periods: 1 month,^37,50,52^ 3 months,^48,55^ 6 months,^37,49,51^ or 12 months.^37–40,42,45,47,53^ The study samples of the eligible studies ranged from 190 participants in Jordan^37^ to 22 473 participants in Chile.^48^ Eight studies were secondary data analysis of population-based surveys^46,49,52,55^ or nationally representative surveys,^38,47,48,56^ and 11 studies were based on face-to-face household or community surveys.^37,39,40–45,50,51,53^ One study was an institutional-based survey.^54^ The associations found between SES indicators and PHC utilization among older adults are summarized in Figure 3.

### Income

In total, we identified 16 studies that investigated the association between income and utilization of PHC, using a wide variety of measures of individual and household current income (Table S4). Fourteen studies reported correlations between income and PHC utilization, and two studies—from Jordan^37^ and Brazil^49^—found no association between income and PHC utilization. For the studies using multivariable analyses methods, most findings (6 of 11 studies) suggested that older people with a higher income had a higher likelihood of using PHC services in Chile^48^; Dominican Republic, Puerto Rico, urban areas of Peru, China, and India^55^; Ethiopia^54^; Estonia^53^; and China,^39,45^ after accounting for confounders, including sociodemographic and illness-related factors. Authors from Brazil^46^ reported a significant link (at the 1% level) between family income per capita and PHC utilization. However, correlations were absent in multivariable findings from China^44^ and Brazil.^49^

Additionally, an inverse association was identified in multivariable analyses in three studies including Cuba (mixed result across countries, within a multicountry study),^55^ Brazil,^47^ and China.^41^ Two of these studies adjusted for participants’ chronic conditions; the findings from Cuba^55^ suggested that older adults with higher household assets were less likely to use primary care in the 3 three months; and similarly, the study from Brazil^47^ reported that older people in the lowest two household wealth quintiles were more likely to have made general practitioner (GP) visits in the past 12 months, but there was no association between income and total number of GP visits. Authors of a study from China^41^ reported that compared to those with lower annual per capita income, older people living in a household with higher annual per capita income had a lower likelihood of using basic public health services. A study from Thailand reported a pro-poor estimate of dental services utilization among older Thais (concentration index = −0.08).^38^ Among those seven pro-rich findings (Figure 3), six studies showed mixed results across different study exposures: study country settings,^55^ types of services and gender,^48^ level of monthly income per family member,^53^ or level of older people's monthly income,^39,45^ or only for family income per capita but not for individual income.^46^

### Education

A total of 14 studies reported an association between education and PHC utilization, with 10 significant findings^41,42,44,46,49,51–55^ (Table S5). Four studies with adjusted results suggested that well-educated older people were more likely to use PHC services compared to less-educated people in Ethiopia,^54^ Brazil,^46^ Estonia,^53^ Cuba, and Nigeria (within a multicountry study).^55^ Additionally, three studies reported inverse associations, which suggested that older people with a lower level of education were more likely to use PHC services in China^41,44^ and in Brazil.^49^ There were four studies where authors found no association, carried out in Jordan,^37^ China,^40,45^ and Chile,^48^ and, within a multisite study, no association was found in Dominican Republic, Puerto Rico, Venezuela, Peru, Mexico, China, and India.^55^

### Employment/Occupation

As shown in Table S6, authors from 3 out of 7 studies reported significant associations between current employment status or previous occupation and PHC utilization. Among the four studies comparing being unemployed/inactive/retired with being currently employed,^37,42,48,54^ two studies showed an association between older people's current employment status and PHC utilization.^42,48^ In Chile, Martinez (2014)^48^ reported that, after adjusting for sociodemographic and disease-related confounders, unemployed or economically inactive older women were more likely to use PHC services. Among male participants, those who were economically inactive were more likely to make preventive visits, while there were no significant associations among currently unemployed older men. A study from China^42^ showed an unadjusted association between current employment status (employed-farming/retired-still working/retired/unemployed-never worked before) and utilization of community health services in the past year.

For the three studies comparing past occupation and PHC utilization,^43,45,46^ the links between previous occupation and older people's PHC utilization are absent in most studies. Only one study from China^43^ suggested that previous work in formal sectors is associated with increased PHC utilization by a multivariable method, but the result was mixed across types of services, in that compared to other types of employees, older employees in public servant roles or institutions and enterprise employees were more likely to use some essential public health services, such as health checkups and lifestyle guidance, compared to employees in other sectors. However, no significant findings were found for using services including health records, health education services, and influenza vaccination. Another study from China^45^ found no correlation between occupation and PHC use, in that occupation was excluded in the multivariable model. A study from Brazil^46^ reported no significant links between economic sector or occupation and PHC use.

### Health Insurance

We identified 7 studies with estimates of the association between enrollment in health insurance plans and PHC utilization (Table S7). A total of 5 of these reported that older people with health insurance were more likely to use PHC services,^40,42,44,55,56^ while 2 studies, 1 from Jordan^37^ and 1 from China,^45^ showed no significant association between insurance and utilization. A study across 9 LMICs showed that older people with health insurance were more likely to use community health services in the past three months across all Latin American and Asian sites, with the exception of rural Peru, rural China, and urban India.^55^ Similarly, in their multivariable analyses, investigators from another multisite study carried out in China, Ghana, India, Mexico, and South Africa^56^ found that insured older people had a higher likelihood of using basic chronic care, with the exception of South Africa. A study carried out in China that compared utilization among older people with different types of health insurance found that those who self-funded were less likely to access PHC services.^42^ Finally, Lu and colleagues (2015)^44^ reported that older people with experience of reimbursed insurance were more likely to use different types of community health services, such as chronic disease management and health examination.

## Discussion

Relative higher economic status—indicated in our systematic review by better access to education, higher income, being unemployed and economically inactive in older age, or having worked in formal sectors and enrollment in health insurance plans—was generally correlated with PHC utilization among older people in LMICs. SDG-3 targets of Health for All and UHC goals are unlikely to be met while this disparity remains. Our review provided some grounds for optimism. Results from Cuba (within a multicountry study),^55^ China,^41,44^ Thailand,^38^ and Brazil^47,49^ indicate pro-poor findings, with older people with lower household wealth or annual per capita income and less education having a higher likelihood of using PHC services.

Consistent with the results of UHC monitoring, Cuba, China, Brazil, and Thailand achieved better in UHC service coverage compared to most of the included countries in this review: In Cuba,^55^ China,^41^ and Thailand,^38^ the captured PHC services are available free of charge. Studies from Brazil^47,49^ covered use of free services available from public sectors. While many LMICs’ health systems have so far failed to deliver PHC that is accessible to all population groups, these studies come from countries that have made recent rapid progress. For example, the Cuban PHC system has successfully established polyclinics, family doctor and nurse programs, which have led to remarkable progress in achieving the WHO health goals for developing countries by 2000. Cubans have a high life expectancy and its health indicators are close or equal to developed countries.^57,58^ In Brazil, the introduction of community-based primary care (Family Health Strategy) has improved health equity by focusing on poorer citizens,^23,59^ primarily funded through taxes.^47^ Since 2002, the implementation of the UHC policy in Thailand has achieved progress in improving the equity of essential health services coverage, resulting in increases in life expectancy and reduced out-of-pocket health expenditures.^60^ Similarly, China has made progress in enhancing the PHC system by increasing government investments^61^ and implementing the National Basic Public Health Service Programme. The Chinese PHC system consists of generalist clinical care and basic public health services, and the basic public health service program provides a set of free services for all residents^62^ that have, to some extent, reduced the disease burden for the poor and improved the equity of health care utilization.^63^ All these government programs may also contribute to the benefits of equally accessing PHC and help to break the “wealth–health” association.^64^ Cost of health services is a major barrier of accessing health care in LMICs. Achievements under free health programs or PHC systems targeted for poor people in Brazil, China, Cuba, and Thailand provide examples for improving equalities.

Our findings suggest that early-life exposures have an influence on PHC utilization in later life. Links exist between education and PHC utilization, with older people with higher education more likely to use PHC. One possible mechanism for the correlation between education and inequalities in PHC utilization may be health literacy.^65^ For example, findings from the Netherlands suggested that health literacy mediated the association between education and out-of-hours primary care services use.^66^ Older people, particularly those with a lower educational level, often have a lower level of health literacy. Limited health literacy restricts access to health information and the ability to make healthy choices, subsequently reinforcing socioeconomic health inequalities.^67^ There is also growing evidence that the effect of early-life socioeconomic conditions may depend on interactions with other risk factors in later life.^25^ An assumption is that early-life exposures, such as education, affect middle- and late-life SES indicators such as income and employment.^68^ Therefore, education may reflect both the long-term influences of early-life socioeconomic exposure itself as well as the cumulative influence of middle- and late-life indicators on late-life health.^69^

We found that exposures later in life have an impact on PHC utilization in older age. Generally, PHC utilization was more likely among older people with higher income. Those enrolled in health insurance plans were also more likely to use PHC. Being economically inactive in old age is related to PHC utilization, but the links with previous occupation are absent. Although the links between unemployment and adverse health outcomes are documented, the effect may be modified through other SES indicators (eg, poverty).^70,71^ The mechanism of how employment status in retired age influences health status is unclear.^72^ The classification and assignment of occupation are differently defined across studies and settings and weakly captured, especially for the retired population.^69^ Late-life income may be affected by the association of pensions with formal employment,^73^ thereby influencing PHC utilization.

We applied a comprehensive search strategy across a wide range of databases to ensure inclusivity. Although our review identified a correlation between socioeconomic exposures and PHC utilization, the design of included studies did not facilitate explanation of the pathways that underlie these relationships. First, all the studies included in this systematic review have a cross-sectional design, meaning that temporal sequence and causality cannot be ascertained. Reverse causality cannot be ruled out as even early-years exposures rely upon recall. We know that exposure to socioeconomic adversity over the life-course is cumulative; so, there is a mismatch between the type of data collected and the nature of the problem. We were not able to analyze interactions between economic exposures in our analyses. The primary objectives of most of the studies included was not to investigate the association between SES and older people's PHC utilization, but to estimate the equality of or the use of PHC services and its correlators in old age. Finally, unmeasured confounders are likely to have had an effect on estimation of correlates. In this systematic review, most eligible studies have taken account of chronic conditions and multimorbidity in their multivariable analyses. However, reviewed studies mostly captured utilization of PHC services by retrospective self-reported binary measure, and we are not able to separate older people who have needs/no needs for PHC services in our estimations of PHC utilization. A conventional assumption is that PHC utilization is correlated with improved health outcomes,^74^ but the opposite is also theoretically true. Older people's health status and their chronic care need influence their decision-making on seeking PHC services.^75^

Our findings suggest that exposure to economic adversity in early and mid-life may not have to lead to inequalities in PHC utilization in older age. We identified studies from Cuba, Brazil, Thailand, and China that appeared to be examples of the success of reforms to social protection programs, financing, payment, and reimbursement mechanisms designed to promote equity. Older people who had health insurance were more likely to use PHC. The vital role that social protection system plays in the prevention of catastrophic health expenditure has been highlighted in developing countries.^76,77^ Some evidence suggests that government protection policies, such as social pensions, can improve the social status of older people and subsequently contribute to improving their health and access to care.^78^ However, previous evidence has pointed out that inequities in enrollment in social protection systems exist for the poor in LMICs. In Senegal and Ghana, poorer older people are less likely to enroll in social health protection programs, even if programs are targeted at improving accessing health care services among poor older people.^79^ Similarly, income has an impact on paying the small premium for enrolling in China's Cooperative Medical System in rural China, and richer people benefit more from the enrollment.^80^ This finding has been replicated among rural, older Ghanaians.^26^ Given the findings of the interplay between the inequalities derived from individual SES indicators and limited PHC utilization at the macro level, the performance of PHC platforms in delivering accessible, good-quality, and needs-driven services may independently hinder individuals’ service use or interact with micro-level socioeconomic inequalities. Due to the limited evidence available, the underlying mechanisms of the association between socioeconomic inequalities and PHC use is still unclear. Nevertheless, more policy inputs are needed to facilitate PHC access among older populations living in societies that are in the process of strengthening their PHC platforms. This will contribute to enhance further integrational and cooperative work with secondary and tertiary care facilities, thereby fulfilling the diverse health needs present in older age.

## Conclusions

Overall, we found inequities in the utilization of PHC across a range of SES indicators among older people from LMICs, relating to different points in the life course, thereby reflecting the cumulative nature of socioeconomic disadvantage. The implementation of health reforms in some developing countries has, to some extent, improved the equity of PHC health systems and benefit to the poor. However, more efforts are needed to increase inputs to the PHC system in limited-resource settings to ensure the accessibility of PHC among older people regardless of their SES, to ensure services are better equipped to address the management of multimorbidity, and to enable them to meet the diverse health care needs that are characteristic of older age. All the articles we identified were cross-sectional studies. Studies are needed that are able to investigate the longitudinal mechanism of SES, care needs, and PHC utilization in older age. Future research should also explore experiences of accessing PHC, including differences by SES, to explain mechanisms for associations, so that interventions can be designed to address these. Although there were notable exceptions, this systematic review suggests a pro-rich phenomenon in PHC use, which highlights the need to promote health equality and prevent the circle of disease and poverty. There is a need to understand and remove barriers to improving accessibility of PHC to older people in LMICs. This will need to be addressed if UHC and SDG3 are to be met.

## Supplemental Material

sj-docx-1-joh-10.1177_00207314211041234 - Supplemental material for Inequalities in Older age and Primary Health Care Utilization in Low- and Middle-Income Countries: A Systematic Review Click here for additional data file. Supplemental material, sj-docx-1-joh-10.1177_00207314211041234 for Inequalities in Older age and Primary Health Care Utilization in Low- and Middle-Income Countries: A Systematic Review by Qian Gao, A. Matthew Prina, Yuteng Ma, David Aceituno and Rosie Mayston in International Journal of Health Services
