# Supplementary material for: Inequalities in Older age and Primary Health Care Utilization in Low- and Middle-Income Countries: A Systematic Review
Source: Int J Health Serv. 2021 Oct 21;52(1):99–114. doi: 10.1177/00207314211041234 (PMC8645300; doi:10.1177/00207314211041234)
Supplement: sj-docx-1-joh-10.1177_00207314211041234 - Supplemental material for Inequalities in Older age and Primary Health Care Utilization in Low- and Middle-Income Countries: A Systematic Review [file sj-docx-1-joh-10.1177_00207314211041234.docx]

**S1 Table**

**Study Eligibility Criteria**

|  | **The inclusion criteria** | **The exclusion criteria** |
| --- | --- | --- |
| **Type of research** | Quantitative research where indicators of SES and use of PHC were measured and reported. | Studies that did not measure and report indicators of SES and PHC, or studies that only included SES or PHC utilization, were also excluded. |
| **Study design** | Any primary quantitative research (e.g., randomized controlled trials studies, quasi-experimental studies, cross-sectional studies, cohort studies, case-control studies, and longitudinal studies), not necessary population-based studies. | Studies were excluded if they are comments, editorials, case reports, case series, qualitative studies, mixed method studies, or systematic reviews. |
| **Publication** | Be published on peer-reviewed (and electronic) journals, no restriction on publication date. | None |
| **Language** | No language restriction was applied. | None |
| **Study population** | The mean age should be 60 years old inclusive, according to WHO’s definition of elderly. If the study was not exclusively looking at this age group, it had to present data in an age-stratified manner to allow for extraction of the specific age-related results. | Report results from studies carried out among people aged less than 60 years. |
| **Study settings** | Studies carried out in LMICs that correspond to the World Bank country classification in the year of study conduction. | Studies carried out in high-income country settings. |
| **Outcomes** | Use of PHC, including using services in PHC platforms (including community-based care, health centers, first-level hospitals, population-based interventions). In which, community health care includes community-based rehabilitation and community-based inclusive development programme (health-related). Government PHC provided by general primary care workers, like general practitioners (GPs), non-specialist physician and traditional healers were also included. Primary services also include case identification, dental services, basic evidence-based interventions and basic referral pathways to secondary care. | Others |
| **Exposures** | The socioeconomic status (SES): the established vertical status indicators (education, income, and employment) and the insurance status. As well as the economic domain includes social class, poverty, occupation, income inequality, deprivation, assets index. | Others |
| **Measurement of SES** | There were no restrictions on the measure of SES eligible for inclusion; relevant SES measured by economic domains were included. | Studies that did not measure and report indicators of socioeconomic status. |
| **Measurement of study outcomes** | Studied that measured PHC utilization by both self-reported and standardized measure tools were included. | Study was conducted with utilization of health services from specialists in secondary or tertiary hospitals and informal (home/unpaid) care. |
|  | Studies in which primary outcome was another factor but included both SES and primary health service usage as covariates or latent factors were also included. | Studies only included SES or the utilization of PHC were also excluded. |

**S2 Table**

**Aggregated results of JBI critical appraisal**

| **Authors, Year** | **Inclusion** | **Study subjects and setting** | **Exposure measurement** | **Measurement of the condition** | **Confounders Measurement** | **Strategies to deal with confounding factors** | **Outcomes measurement** | **Appropriate statistical analysis** |
| --- | --- | --- | --- | --- | --- | --- | --- | --- |
| Alkhawaldeh et al., 2014 | High | High | High | High | High | High | Moderate | High |
| Albanese et al., 2011 | High | High | High | High | High | High | High | High |
| Ayele et al., 2017 | High | High | High | High | High | High | Moderate | High |
| Bos et al., 2007 | High | High | High | High | High | High | Low | High |
| Goeppel et al., 2016 | High | High | High | High | High | High | High | High |
| Macinko et al., 2018 | High | High | High | High | High | High | High | High |
| Martinez, 2014 | High | High | High | High | High | High | Moderate | High |
| Polluste et al., 2009 | Moderate | High | High | High | High | High | Moderate | High |
| Rodrigues et al., 2009 | High | High | High | High | High | High | Moderate | High |
| Somkotra et al., 2013 | Moderate | High | High | High | High | Moderate | High | Moderate |
| Wang et al., 2012 | High | High | Moderate | Moderate | High | High | Moderate | High |
| He et al., 2013 | Moderate | High | Moderate | Moderate | High | High | Moderate | High |
| He et al., 2012 | Moderate | High | Moderate | Moderate | High | High | Moderate | High |
| Sun et al., 2013 | Moderate | High | High | Moderate | High | High | Moderate | High |
| Wen et al., 2015 | High | High | Moderate | Moderate | High | High | Moderate | High |
| Lu et al., 2015 | High | High | High | Moderate | High | High | Moderate | High |
| Xi et al., 2010 | Moderate | High | Moderate | Moderate | High | High | Moderate | High |
| Melguizo-Herrera & Castillo-Ávila, 2012 | High | High | Moderate | High | High | High | Moderate | Moderate |
| Paskulin et al., 2011 | High | High | Moderate | High | High | Low | Low | Low |
| Rodrigues et al., 2008 | High | High | High | High | High | High | Low | Moderate |

**S3 Table**

**Measures of SES and PHC utilization from selected studies**

| **Authors, year** | **Description of SES (Examples of studies using this measure)** | **Description of PHC utilization (Examples of studies using this measure)** |
| --- | --- | --- |
| Alkhawaldeh et al., 2014 | Employment status (unemployed, retired and employed); Education level (no education/primary school education/secondary and higher education); Enabling factors included monthly income (Jordanian Dinars per month) and Health insurance coverage (insured or not insured). | Participants were asked the following: “Did you visit the primary health care center in your region during the past month/past 6 months/past 12 months?” (Yes/No) |
| Albanese et al., 2011 | Educational level (no education/some, completed primary/completed secondary/completed tertiary); Wealth was assessed according to the number of reported household assets (motor vehicles; television; fridge and/or freezer; water and electricity utilities; telephone; plumbed toilet; plumbed bathroom); Health insurance (participants were asked to disclose whether they had purchased any medical insurance cover) | Community health care services utilization was defined as any reported use in the three months prior to the survey of government primary care, hospital outpatient, private doctor, traditional healer, and other community medical services. (Yes/No) |
| Ayele et al., 2017 | Educational status (unable to read and write, primary, secondary and tertiary education); Average monthly income (in USD) (<100/100–150/>150); Employment status (self-employed/government-employed/unemployed) | Complementary and alternative medicine utilization: Use of five CAM modalities, including traditional herbal remedies, special foods (e.g., honey, black seed, ginger, or others), a variety of dietary supplements and spiritual healing (prayers, fasting, lighting candles, consuming holy water). (Yes/No) |
| Bos et al., 2007 | Education (no formal education, including illiterate/primary school/secondary school/university education); Economic sector (1=worked on agriculture during most of life/0=other); Occupation (1=non-specialized occupation during most of life/0=other); Individual income of the elderly (Logarithmic transformation from Currency of Brazil-reais); Household income per capita (Logarithmic transformation from reais), | Use of primary care (Yes/No) The survey questions regarding Health care utilization addressed the place of first contact with health care services (primary care). The most common public providers were public ambulatory clinics and doctor’s offices that provide services through SUS. In this paper, the term “ambulatory clinic” refers to outpatient facilities (no overnight admission/care). The most common private providers were independent, private doctor’s offices. |
| Goeppel et al., 2016 | Health insurance (insured: a person with any type of health insurance was classified as insured/uninsured) | Access to basic chronic care was assessed using a compound indicator with three components: (i) the provision of treatment, such as medications or advice on physical activity or diet, for each of the patient’s conditions; (ii) visiting outpatient services for the chronic condition or conditions one or more times in the past reported year; and (iii) maintenance of a stable health state after the past outpatient visit. In the surveys, health care providers were categorized as medical doctors, nurses, physiotherapists or traditional practitioners. (Yes/No) |
| Macinko et al., 2018 | Household wealth quintiles (Being in the lowest quintiles of a household wealth score (from a principal component analysis of a list of 16 common household goods)) (Lowest 2 quintiles/higher quintiles) | Self-reported number of any general practitioner or non-specialist doctor visits in the past 12 months. (None/1 or more GP visit, past year) & (Mean of GP visits) |
| Martinez, 2014 | Income deciles (Decile of per capita household income (measured in 2011 Chilean pesos); Education (primary or less, secondary, college and above); Employment status (employed, unemployed and inactive) | Had at least one preventive visit of preventive visits/acute care visits (Primary care visits for acute health problems) in the past three months. (Yes/No) |
| Polluste et al., 2009 | Education (elementary, secondary and higher), Income per family member per month (<EEK 2000 (<EUR 127.8)/EEK 2001–3000 (EUR 127.9–191.7)/>EEK 3000 (>EUR 191.7)) | “Have you used the following health services during the previous 12 months: general practitioner (GP), outpatient specialist, dentist, inpatient services, and/or ambulance services and if so, how many times?” (Yes/No) |
| Rodrigues et al., 2009 | Level of schooling (0 year/1 to 4 years/5 to 8 years/9 or more years); Family income (0-250 Reais/251-450 Reais/451-600 Reais/601-915 Reais/916-4800 Reais) | Use medical visits at the UBS (PHC unit) in the past six months (Yes/No) |
| Somkotra et al., 2013 | Household assets index (poorest quintile (Q1)/2^nd^ quintile (Q2)/3^rd^ quintile (Q3)/4^th^ quintile (Q4)/richest quintile (Q5)) | Oral health care utilizations in the 12 months preceding the surveys (Yes/No) |
| Wang et al., 2012 | Monthly income (≤500/500-1000/1001-1500/1501-2000/2000+ RMB) | Use community health services during the past year (Yes/No) |
| He et al., 2013 | level of education (illiteracy/primary/secondary school/high school/post-secondary/professional school/university and above); Health insurance (Yes/No) | Use community health services during the past year (Yes/No) |
| He et al., 2012 | Educational attainment (less than primary education/secondary education/high school and above), Annual per capita income (<5000 RMB/5000-10000/>10000RMB) | Basic public health service (health checkup) (Yes/No) |
| Sun et al., 2013 | Health insurance (self-fund/basic medical insurance for urban workers/free medical treatment/new rural cooperative medical insurance/others), level of education (secondary school and below/high school and post-secondary/professional school and above), employment status (employed- farming)/retired- still working)/retired/unemployed-never worked before), household income (<1000/1000-/2000-/5000 and above RMB) | Whether community health services have been used in the past year (Yes/No) |
| Wen et al., 2015 | Occupation (others/employees in public servants or institutions/enterprise employees) | Use essential public health services (health records (Yes/No)/health education services (Usually or occasionally/little or never)/free checkup (Yes/No)/influenza vaccination (Yes/No)/lifestyle guidance (Yes/No) |
| Lu et al., 2015 | Education (illiterate/primary school/middle school or high school/colleges and above); Monthly income (<500/501-2000/2001-3999/>4000 RMB); Health insurance (Whether the type of medical insurance can be reimbursed) (Yes/No) | The utilization of community health services (Outpatient diagnosis and treatment of common diseases/Prevention and control services of Chronic diseases/Chronic disease management services/Health records management services/Family visit service/Health checkup services/Medication guidance service/Dietary guidance services/Health education and counselling services) (Yes/No) |
| Xi et al., 2010 | Education (illiterate/primary school/middle school/colleges and above); Monthly income (<300/300-/500-/800 and above RMB); Health insurance (self-fund/urban basic health insurance/free medical treatment/other social health insurance); Occupation (before 60 years old) (managers/technicists/worker/farmer/unemployment or part-time employment) | The utilization of community health services during the past year (Yes/No) |
| Melguizo-Herrera & Castillo-Ávila, 2012 | SES was defined according to housing status and income; only low and middle SES were included | Participants were asked if they have visited the primary health care center in their region during the past month? (Yes/No) |
| Paskulin et al., 2011 | Education level (incomplete primary, complete primary, high school/superior) | Participants were asked if they have visited the primary health care center in their region during the las 6 months? (Yes/No) |
| Rodrigues et al., 2008 | Education (Years of education: 0 year/1-4 years/5-8 years/9 years and above); Monthly family income (poorest quintile (Q1)/2^nd^ quintile (Q2)/3^rd^ quintile (Q3)/4^th^ quintile (Q4)/richest quintile (Q5)) | Participants were asked if they have visited the primary health care center in their region during the past month? (Yes/No) |

**S4 Table. Income and PHC utilization among older adults in LMICs**

| **Income types** | **Authors, Year** | **Statistical information Analysis** | **Evaluated confounders** | **Outcome** | **Crude effect size** | **Adjusted effect size; 95% confidence intervals (CIs)** |
| --- | --- | --- | --- | --- | --- | --- |
| Monthly income (Jordanian Dinars per month) | Alkhawaldeh et al., 2014 | Pearson’s Correlation | Age, education, employment status, tobacco use, chronic illnesses, perceived general health today, physical component summary (PCS) score of SF-12v2 | PHC Utilization in the past 1 month | (r = -0.135)-NS | NA- Excluded in the model |
|  |  |  | Age, education, employment status, tobacco use, chronic illnesses, perceived general health today, self-perceived general health in the past 6 months | PHC Utilization in the past 6 months | (r = -0.073)-NS | NA- Excluded in the model |
|  |  |  | Age, education, employment status, tobacco use, chronic illnesses, perceived general health today, self-perceived general health in the past 12 months | PHC Utilization in the past 12 months | (r = -0.111)-NS | NA- Excluded in the model |
| Wealth (number of household assets) | Albanese et al., 2011 | Poisson Regression | Age, gender, educational level, number of assets, health insurance, currently married, living with children, ICD-10 depression, dementia, number of physical illnesses | Use of any community health care services in the past three months | NA | Ref. Lower, Cuba (aPR=0.91, 95%CI 0.84-0.98);  Dominican Republic (aPR=1.11, 95%CI 1.01-1.22); Puerto Rico (aPR=1.09, 95%CI 1.03-1.15);  Venezuela (aPR=1.06, 95%CI 0.98-1.14);  Peru urban (aPR=1.28, 95%CI 1.14-1.44);  Peru rural (aPR=1.04, 95%CI 0.78-1.38);  Mexico urban (aPR=0.95, 95%CI 0.87-1.03);  Mexico rural (aPR=1.02, 95%CI 0.92-1.12);  China urban (aPR=1.34, 95%CI 1.16-1.55);  China rural (aPR=0.57, 95%CI 0.32-1.03);  India urban (aPR=1.44, 95%CI 1.28-1.62);  India rural (aPR=0.93, 95%CI 0.84-1.02);  Nigeria-Not assessed; Meta-analytical pooled effect (aPR=1.08, 95%CI 1.00-1.17) |
| Average monthly income (in USD) <100/100-150/>150 | Ayele et al., 2017 | Univariate Analysis and Multivariate Logistic Regression | Gender, residence, educational status, presence of co-morbidity | Complementary and alternative medicine utilization | P = 0.013 | Ref. Average monthly income < 100 USD; 100-150 USD (OR = 1.57, 95%CI 1.32-4.42); >150 USD (OR = 2.46, 95%CI 1.52-5.82) |
| Individual income of the elderly (Logarithmic transformation) | Bos et al., 2007 | Heckman-Probit Regression | Age, sex, race, education, marital status, economic sector, occupation, family size, family income per capita (log), morbidity scale, total ambulatory clinics per capita, total physicians per capita, public ambulatory clinics per capita, private physicians per capita | Use of primary care | NA | Marginal effect = 0.002; SE = 0.003 (NS) |
| Household income per capita (Logarithmic transformation) |  |  |  | Use of primary care | NA | Marginal effect = 0.029; SE = 0.008 (Significant at the 1% level) |
| Household wealth quintiles (lowest 2/higher quintiles) | Macinko et al., 2018 | Poisson Regression | Age, sex, educational attainment, civil status, and region of the country, individual’s type of health care coverage, primary care problems, the presence of any previously diagnosed chronic conditions, any functioning limitations, household wealth score (from a principal component analysis of a list of 16 common household goods). | Any GP visit in the past 12 months | NA | Ref. Higher quintiles (Lowest 2 household wealth quintiles PR = 1.27, 95%CI 1.06-1.53) |
|  |  |  |  | Number of GP visits in the past 12 months | NA | Ref. Higher quintiles (Lowest 2 household wealth quintiles PR = 1.02, 95%CI 0.90-1.15) |
| Income deciles (Decile of per capita household income) | Martinez, 2014 | Logistic Regression | Age, gender, ethnicity, living in a rural zone, education, employment status, perceived health status, type of health insurance | Preventive visits in the past three months | NA | Female: Ref. 1 income decile; 2 decile (OR = 1.2, SE = 0.26)(NS); 3 decile (OR = 1.6, SE = 0.33)(P<0.05); 4 decile (OR = 1.4, SE = 0.28)(NS); 5 decile (OR = 1.7, SE = 0.36) (P<0.01); 6 decile (OR = 2.0, SE = 0.41) (P<0.01); 7 decile (OR = 1.7, SE = 0.37) (P<0.05); 8 decile (OR = 1.6, SE = 0.36) (P<0.05); 9 decile (OR = 1.3, SE = 0.33)(NS); 10 decile (OR = 1.6, SE = 0.33) (P<0.05);  Male: Ref. 1 income decile; 2 decile (OR = 1.0, SE = 0.19); 3 decile (OR = 1.2, SE = 0.22); 4 decile (OR = 1.1, SE = 0.20); 5 decile (OR = 1.2 SE = 0.20); 6 decile (OR = 1.2, SE = 0.22); 7 decile (OR = 1.0, SE = 0.20); 8 decile (OR = 1.1, SE = 0.21); 9 decile (OR = 1.2, SE = 0.24); 10 decile (OR = 1.3, SE = 0.28) (All-NS) |
|  |  |  |  | Acute care visits in the past three months |  | Female: Ref. 1 income decile; 2 decile (OR = 0.9, SE = 0.22); 3 decile (OR = 1.1, SE = 0.26); 4 decile (OR = 1.2, SE = 0.27); 5 decile (OR = 1.2, SE = 0.26); 6 decile (OR = 1.1, SE = 0.26); 7 decile (OR = 1.5, SE = 0.36)(P<0.1); 8 decile (OR = 1.5, SE = 0.36)(P<0.1); 9 decile (OR = 1.2, SE = 0.34); 10 decile (OR = 1.4, SE = 0.41)); (Others NS  Male: Ref. 1 income decile; 2 decile (OR = 1.2, SE = 0.26); 3 decile (OR = 0.8, SE = 0.18); 4 decile (OR = 0.8, SE = 0.18); 5 decile (OR = 1.0, SE = 0.21); 6 decile (OR = 0.6, SE = 0.14)(P<0.05); 7 decile (OR = 0.9, SE = 0.20); 8 decile (OR = 1.1, SE = 0.24); 9 decile (OR = 0.8, SE = 0.19); 10 decile (OR = 0.8, SE = 0.20); (Others-NS) |
| Income per family member per month (<EEK 2000 (<EUR 127.8)/EEK 2001-3000 (EUR 127.9-191.7) />EEK 3000 (>EUR 191.7)) | Polluste et al., 2009 | Binary Logistic Regression | Age, gender, native language, education, place of residence, self-reported health status and presence of chronic illness, having a family member with a chronic illness, and the simplicity of access to a GP | Use of health services (GPs/dentists) during the previous 12 months | NA | **Visit GPs:** Ref. <EEK 2000 (<EUR 127.8); EEK 2001–3000 (EUR 127.9–191.7) OR = 7.2, 95%CI 1.1-45.5; >EEK 3000 (>EUR 191.7) OR = 3.1, 95%CI 0.7-13.5.  **Visit dentists:** Ref. <EEK 2000 (<EUR 127.8); EEK 2001–3000 (EUR 127.9–191.7) OR = 4.5, 95%CI 1.0-20.8; >EEK 3000 (>EUR 191.7) OR = 1.1, 95%CI 0.4-2.8 |
| Family income (0-250 Reais/251-450 Reais/451-600 Reais/601-915 Reais/916-4800 Reais) | Rodrigues et al., 2009 | Poisson Regression | Age, schooling, functional disability, care model of the UBS | Use of medical visits at the PHC unit in the past six months | NA | Southern Brazil (n = 1332): Ref. 0-250 reais; 251-450 reais PR = 0.88 (95%CI 0.71-1.10); 451-600 reais PR = 1.08 (95%CI 0.92-1.26); 601-915 reais PR = 0.97 (95%CI 0.82-1.14); 916-4800 reais PR = 0.82 (95%CI 0.66-1.01);  Northeastern Brazil: NA |
| Household assets index (1-5 quintile) | Somkotra et al., 2013 | Concentration Index (CI) | NA | Oral health care utilization in health center or community hospital in the 12 months preceding the surveys (2003) | Poorest (Q1) = 3.3%; Q2 = 4.3%; Q3 = 4.3%; Q4 = 3.0%; Q5 = 2.4%; population average = 3.5%  Pearson χ^2^ test (P<0.001) | The concentration index (CI) = -0.08, (P<0.05) |
|  |  |  |  | Oral health care utilization in health center or community hospital in the 12 months preceding the surveys (2009) | Poorest (Q1) = 3.9%; Q2 = 4.7%; Q3 = 3.5%; Q4 = 3.9%; Q5 = 3.6%; population average = 3.9%  Pearson χ^2^ test (P<0.001) | The concentration index (CI) = -0.08, (P< 0.05) |
| Monthly income (≤500/500-1000/1001-1500/1501-2000/2000+ RMB) | Wang et al., 2012 | Logistic Regression | Age, sex, ethnicity, educational level, occupation, family type, methods of medical expenses payment, chronic diseases, awareness of community health services | Use of community health services during the past year | NA | Ref. ≤500 RMB; 500-1000RMB (OR = 5.119, 95%CI 1.458 -17.977); 1001-1500 RMB (OR = 0.675, 95%CI 0.381-1.605); 1501-2000 RMB (OR = 0.487, 95%CI 0.341-1.767); 2000+ RMB (OR = 0.139, 95%CI 0.154-1.256) |
| Annual per capita income (<5000 RMB/5000-10000/>10000RMB) | He et al., 2012 | Logistic Regression | Sex, educational attainment, geographic accessibility, health knowledge, awareness of health service | Use of basic public health service (health checkup) | Ref. <5000 RMB, (OR = 0.81, 95%CI 0.68-0.97) | Ref. <5000 RMB; Higher income (OR = 0.74, 95%CI 0.62-0.89) |
| Household income (<1000 /1000-/2000-/5000 and above RMB) | Sun et al., 2013 | Chi-Square Test | Urban and rural areas, age, marital status, level of education, occupation, health insurance, living status, chronic disease conditions, self-care dependency | Use of community health services in the past year | χ^2^ = 17.719, P = 0.001 | NA |
| Monthly income (<500/501-2000/2001-3999/>4000 RMB) | Lu et al., 2015 | Logistic Regression | Gender, education, living status, whether health insurance could cover certain of expenses, chronic diseases, willingness of seeking treatments in community when illness | Use of community health services | (1) Outpatient diagnosis and treatment of common diseases (χ^2^ = 40.18, P<0.001); (2) Prevention and control services of Chronic diseases (χ^2^ = 8.0, P = 0.05); (3) Chronic disease management services (χ^2^ = 7.28, P = 0.06); (4)Health records management services (χ^2^ = 11.42, P = 0.01); (5) Family visit service (χ^2^ = 21.30, P<0.001); (6)Home visits (χ^2^ = 8.48, P = 0.04); (7) Health checkup services (χ^2^ = 49.50, P<0.001); (8) Medication guidance service (χ^2^ = 19.40, P<0.001); (9) Dietary guidance services (χ^2^ = 13.16, P<0.001); (10) Health education and counselling services (χ^2^ = 16.37, P<0.001) | NS- Excluded in the model |
| Monthly income (<300/300-/500-/800 and above RMB) | Xi et al., 2010 | Logistic Regression | Age, gender, education, health insurance, occupation (before 60 years old), living status, chronic disease, severity of disease, distance to community care center, awareness of community health service | Use of community health services during the past year | NA | Ref. ≤300 RMB; 300-RMB (OR = 2.295. 95%CI 1.055-4.994); 500-RMB (OR = 0.629, 95%CI 0.281-1.405); ≥800 RMB (OR = 0.776, 95%CI 0.341-1.767) |
| SES (measured as housing status and income) | Melguizo-Herrera & Castillo-Ávila, 2012 | Logistic Regression (Chi-Square Test) | Age, gender, education, civil status, chronic disease (yes/no), waiting time | PHC utilization in the past month | (χ^2^ = 4.12, P = 0.043) | Not clear |
| Family monthly income level (1-5 quintile) | Rodrigues et al., 2008 | Univariate analysis (Chi- Square Test) | Gender, age, skin color, marital status, education, smoking, health status perception, daily activities limitations | PHC utilization in the past month | P-value (Southern regions P = 0.002; Northern regions P = 0.001) | Not clear |

** NA: Not available*

*NS: Not significant*

**S5 Table. Educational level and PHC utilization among older adults in LMICs**

| **Education types** | **Authors, Year** | **Statistical information Analysis** | **Evaluated confounders** | **Outcome** | **Crude effect size** | **Adjusted effect size; 95% confidence intervals (CIs)** |
| --- | --- | --- | --- | --- | --- | --- |
| Education level (no education/primary/secondary and higher education) | Alkhawaldeh et al., 2014 | Pearson’s Correlation & Binary Logistic Regression | Age, employment, tobacco use, chronic illnesses, perceived general health today, physical component summary (PCS) score of SF-12v2 | PHC Utilization in the past 1 month | (r = −0.220) | Ref. Secondary and higher education; No education (OR = 1.37, 95%CI 0.37-5.03); Primary education (OR = 0.66, 95%CI 0.24-1.82) |
|  |  |  | Age, employment, tobacco use, chronic illnesses, perceived general health today, self-perceived general health in the past 6 months | PHC Utilization in the past 6 months | (r = −0.200) | Ref. Secondary and higher education; No education (OR = 0.76, 95%CI 0.14-4.00); Primary education (OR = 0.45, 95%CI 0.13-1.51) |
|  |  |  | Age, employment, tobacco use, chronic illnesses, perceived general health today, self-perceived general health in the past 12 months | PHC Utilization in the past 12 months | (r = −0.240) | Ref. Secondary and higher education; No education (OR = 3.26, 95%CI 0.56-18.80); Primary education (OR = 1.50, 95%CI 0.44-5.07) |
| Educational level (no education/some, completed primary/completed secondary/completed tertiary) | Albanese et al., 2011 | Poisson Regression | Age, gender, number of assets, health insurance, currently married, living with children, ICD-10 depression, dementia, number of physical illnesses | Use of any community health care services in the past three months | NA | Cuba (aPR = 1.06, 95%CI 1.02-1.10)  Dominican Republic (aPR = 1.03, 95%CI 0.98-1.08)  Puerto Rico (aPR = 0.99, 95%CI 0.98-1.01)  Venezuela (aPR = 1.00, 95%CI 0.96-1.04)  Peru urban (aPR = 1.05, 95%CI 0.99-1.12)  Peru rural (aPR = 0.98, 95%CI 0.85-1.13)  Mexico urban (aPR = 1.02, 95%CI 0.99-1.06)  Mexico rural (aPR = 1.02, 95%CI 0.96-1.08)  China urban (aPR = 1.04, 95%CI 0.98-1.10)  China rural (aPR = 0.86, 95%CI 0.64-1.16)  India urban (aPR = 1.02, 95%CI 0.98-1.07)  India rural (aPR = 1.03, 95%CI 0.97-1.09)  Nigeria (aPR = 1.14, 95%CI 1.06-1.24)  Meta-analytical pooled effect (aPR = 1.03, 95%CI 1.01-1.05) |
| Educational level (unable to read and write/primary  /secondary/tertiary education) | Ayele et al., 2017 | Univariate Analysis and Multivariate Logistic Regression | Gender, residence, average monthly income, presence of co-morbidity | Complementary and alternative medicine utilization | P = 0.012 | Ref. Unable to read and write; Primary (OR = 1.73, 95% CI 0.41-3.04); Secondary (OR = 2.44, 95%CI 1.62-5.17); Tertiary education (OR = 2.71, 95%CI 1.64-4.51) |
| Educational level (no formal education/primary school/secondary school/university education) | Bos et al., 2007 | Heckman-Probit Regression | Age, sex, race, marital status, economic sector, occupation, family size, individual income (log), family income per capita (log), morbidity scale, total ambulatory clinics per capita, total physicians per capita, public ambulatory clinics per capita, private physicians per capita | Use of primary care | NA | Ref. No formal education, including illiterate; Primary (Marginal effect = 0.011, SE = 0.015)-NS/Secondary (Marginal effect = 0.024, SE = 0.017)- NS/University (Marginal effect = 0.045, SE = 0.024)- (Significant at the 10% level) |
| Education (primary or less /secondary/college and above) | Martinez, 2014 | Logistic Regression | Age, gender, ethnicity, living in a rural zone, employment status, perceived health status, type of health insurance | Preventive visits in the past three months | NA | Female: Ref. Primary; Secondary (OR = 0.9, SE = 0.12); College (OR = 1.0, SE = 0.18) (NS);  Male: Ref. Primary; Secondary (OR = 1.0, SE = 0.13); College (OR = 1.1, SE = 0.24) (NS) |
|  |  |  |  | Acute care visits in the past three months |  | Female: Ref. Primary; (Secondary OR = 1.2, SE = 0.15); College (OR = 0.9, SE = 0.19) (NS);  Male: Ref. Primary (Secondary OR = 0.9, SE = 0.13); College (OR = 0.7, SE = 0.15) (NS) |
| Educational level (elementary/secondary/higher) | Polluste et al., 2009 | Binary Logistic Regression | Age, gender, native language, income and place of residence, self-reported health status and presence of chronic illness, having a family member with a chronic illness, and the simplicity of access to a GP | Visit dentists during the previous 12 months | NA | Visit dentists: Ref. Elementary; Secondary (OR = 3.9, 95%CI 1.2-12.5); Higher (OR = 0.9, 95%CI 0.3-2.7) |
| Level of schooling (0/1 to 4 years/5 to 8 years/9 or more years) | Rodrigues et al., 2009 | Poisson Regression | Age, family income in quintiles, functional disability, care model of the UBS | Use of medical visits at the PHC unit in the past six months | NA | Southern Brazil (n = 1332): Ref. 0 year; 1-4 years (PR = 0.83, 95%CI 0.71-0.96); 5-8 years (PR = 0.81, 95%CI 0.67-0.98); 9 or more years (PR = 0.64, 95%CI 0.41-0.98) |
|  |  |  |  |  |  | Northeastern Brazil (n = 1479): Ref. 0 year; 1-4 years (PR = 0.88, 95%CI 0.76-1.03); 5-8 years (PR = 0.84, 95%CI 0.70-0.99); 9 or more years (PR = 0.40, 95%CI 0.22-0.72) |
| Level of education (illiteracy/primary/secondary school  /high school/post-secondary/professional school/university and above) | He et al., 2013 | Logistic Regression | Age, gender, health insurance, chronic diseases, walking time to community health service center, medical expenses in the latest year | Use of community health services during the past year | Educational level attainment (χ^2^ = 9.214, P = 0.162) | NS |
| Educational attainment (less than primary education/secondary education/high school and above) | He et al., 2012 | Logistic Regression | Sex, annual per capita income, geographic accessibility, health knowledge, awareness of health service | Use of basic public health service (health checkup) | Ref. less than primary education, (OR = 0.56, 95%CI 0.39-0.79) | Ref. less than primary education, higher education attainment (OR = 0.56, 95%CI 0.39-0.80) |
| Educational level (secondary school and below/high school and post-secondary/professional school and above), | Sun et al., 2013 | Chi-Square Test & Logistic Regression | Urban and rural areas, age, marital status, occupation, household income, health insurance, living status, chronic disease conditions, self-care dependency | Use of community health services in the past year | χ^2^ = 10.122, P = 0.006 | NA |
| Educational level (illiterate/primary school/middle school or high school/colleges and above) | Lu et al., 2015 | Logistic Regression | Gender, living status, monthly income, whether health insurance could cover certain of expenses, chronic diseases, willingness of seeking treatments in community when illness | Use of community health services | (1) Outpatient diagnosis and treatment of common diseases (χ^2^ = 56.36, P<0.001); (2) Prevention and control services of Chronic diseases (χ^2^ = 7.67, P=0.02); (3) Chronic disease management services (χ^2^ = 7.52, P=0.02); (4) Health records management services (χ^2^ = 17.04, P<0.001); (5) Family visit service (χ^2^ = 21.04, P<0.001); (6) Home visits (χ^2^ = 4.68, P = 0.10); (7) Health checkup services (χ^2^ = 34.14, P<0.001); (8) Medication guidance service (χ^2^ = 14.73, P<0.001); (9) Dietary guidance services (χ^2^ = 8.36, P = 0.02); (10) Health education and counselling services (χ^2^ = 8.12, P = 0.02) | Use services- Outpatient diagnosis and treatment of common diseases: Ref. lower education, higher education attainment (OR = 0.78, 95%CI 0.63-0.98) |
| Educational level (illiterate/primary school/middle school/colleges and above) | Xi et al., 2010 | Logistic Regression | Age, gender, monthly income(poverty), health insurance, occupation (before 60 years old), living status, chronic disease, severity of disease, distance to community care center, awareness of community health service | Use of community health services during the past year | NA | NS- Excluded in the model |
| Education level (incomplete primary, complete primary, high school/superior) | Paskulin et al., 2011 | Pearson’s Correlation | Not performed | PHC utilization in the past 6 months | P<0.001 | NA |
| Years of education: 0 year/1-4 years/5-8years/9 years and above | Rodrigues et al., 2008 | Univariate Analysis (Chi-Square Test) | Gender, age, skin color, marital status, family income, smoking, health status perception, daily activities limitations. | PHC utilization in the past month | P-value (Southern regions P = 0.134; Northern regions P<0.001) | NA |

** NA: Not available*

*NS: Not significant*

**S6 Table. Employment/Occupation and PHC utilization among older adults in LMICs**

| **Employment/Occupation Types** | **Authors, Year** | **Statistical information Analysis** | **Evaluated confounders** | **Outcome** | **Crude effect size** | **Adjusted effect size; 95% confidence intervals (CIs)** |
| --- | --- | --- | --- | --- | --- | --- |
| Employment status (unemployed, retired, and employed) | Alkhawaldeh et al., 2014 | Pearson’s Correlation & Binary Logistic Regression | Age, education, tobacco use, chronic illnesses, perceived general health today, physical component summary (PCS) score of SF-12v2 | PHC utilization in the past 1 month | (r = 0.158) | Ref. Employed; Unemployed (OR = 1.49, 95%CI 0.11-19.59); Retired (OR = 1.40, 95%CI 0.11-17.40) |
|  |  |  | Age, education, tobacco use, chronic illnesses, perceived general health today, self-perceived general health in the past 6 months | PHC utilization in the past 6 months | (r = 0.178) | Ref. Employed; Unemployed (OR = 1.99, 95%CI 0.20-19.5); Retired (OR = 1.82, 95%CI 0.20-16.1) |
|  |  |  | Age, education, tobacco use, chronic illnesses, perceived general health today, self-perceived general health in the past 12 months | PHC utilization in the past 12 months | (r = 0.218) | Ref. Employed; Unemployed (OR = 2.62, 95%CI 0.23-29.02); Retired (OR = 4.57, 95%CI 0.44-47.19) |
| Employment status (Self-employed/Government-employed/Unemployed) | Ayele et al., 2017 | Univariate Analysis and Multivariate Logistic Regression | Gender, residence, educational status, average monthly income, presence of co-morbidity | Complementary and alternative medicine utilization | P = 0.273 | NA |
| Economic sector (worked on agriculture during most of life/other ); | Bos et al., 2007 | Heckman-Probit Regression | Age, sex, race, education, marital status, occupation, family size, individual income (log), family income per capita (log), morbidity scale, total ambulatory clinics per capita, total physicians per capita, public ambulatory clinics per capita, private physicians per capita | Use of primary care | NA | NS-Marginal effect = -0.012; SE = 0.019 |
| Occupation (non-specialized occupation during most of life/other) |  |  |  | Use of primary care |  | NS-Marginal effect = -0.019; SE = 0.013 |
| Employment Status (employed/unemployed/inactive) | Martinez, 2014 | Logistic Regression | Age, gender, ethnicity, living in a rural zone, education, perceived health status, type of health insurance | Preventive visits in the past three months | NA | Female: Ref. employed; Unemployed (OR = 3.2, SE = 1.73) (P<0.05); Inactive (OR = 1.7, SE = 0.30) (P<0.01)  Male: Ref. employed; Unemployed (OR = 0.9, SE = 0.43) (NS); Inactive (OR = 1.7, SE = 0.17) (P<0.01) |
|  |  |  |  | Acute care visits in the past three months |  | Female: Ref. employed; Unemployed (OR = 1.3, SE = 0.62) (NS); Inactive (OR = 1.0, SE = 0.18) (NS)  Male: Ref. employed; Unemployed (OR = 0.5, SE = 0.23) (NS); Inactive (OR = 1.0, SE = 0.12) (NS) |
| Employment Status (employed- farming/retired- still working/retired/unemployed- never worked before) | Sun et al., 2013 | Chi-Square Test & Logistic Regression | Urban and rural areas, age, marital status, level of education, household income, health insurance, living status, chronic disease conditions, self-care dependency | Use of community health services in the past year | χ^2^ = 14.278, P = 0.003 | NA |
| Occupation (others/employees in public servants or institutions/enterprise employees) | Wen et al., 2015 | Logistic Regression | Gender, health literacy, chronic diseases | Use of essential public health services (health records services) | NA | (1) Ref. others; Employees in public servants or institutions (OR = 1.04, 95%CI 0.81-1.33); Enterprise employees (OR = 1.11, 95%CI 0.76-1.61) |
|  |  |  |  | Use of essential public health services (health education services) (Usually or occasionally /little or never) |  | (2) Ref. others; Employees in public servants or institutions (OR = 0.88, 95%CI 0.65-1.19); Enterprise employees (OR = 1.46, 95%CI 0.96-2.21) |
|  |  |  |  | Use of essential public health services (influenza vaccination) |  | (3) Ref. others; Employees in public servants or institutions (OR = 1.21, 95%CI 0.96-1.54); Enterprise employees (OR = 0.75, 95%CI 0.52-1.08) |
|  |  |  |  | Use of essential public health services (Health checkup) |  | (4) Ref. others; Employees in public servants or institutions (OR = 1.65, 95%CI 1.22-2.23); Enterprise employees (OR = 1.59, 95%CI 1.02-2.47) |
|  |  |  |  | Use of essential public health services (lifestyle guidance) |  | (5) Ref. others; Employees in public servants or institutions (OR = 1.37, 95%CI 1.07-1.74); Enterprise employees (OR = 1.59, 95%CI 1.09-2.33) |
| Occupation (before 60 years old) (managers/technicists/worker/farmer/unemployment or part-time employment) | Xi et al., 2010 | Logistic Regression | Age, gender, education, monthly income (poverty), health insurance, living status, chronic disease, severity of disease, distance to community care center, awareness of community health service | Use of community health services during the past year | NA | NS- Excluded in the model |

** NA: Not available*

*NS: Not significant*

**S7 Table. Health insurance and PHC utilization among older adults in LMICs**

| **Health insurance types** | **Authors, Year** | **Statistical information Analysis** | **Evaluated confounders** | **Outcome** | **Crude effect size** | **Adjusted effect size; 95% confidence intervals (CIs)** |
| --- | --- | --- | --- | --- | --- | --- |
| Health insurance coverage (insured/uninsured) | Alkhawaldeh et al., 2014 | Pearson’s Correlation | Age, education, employment status, tobacco use, chronic illnesses, perceived general health today, physical component summary (PCS) score of SF-12v2 | PHC utilization in the past 1 month | (r = 0.087)-NS | NA-Excluded in the model |
|  |  |  | Age, education, employment status, tobacco use, chronic illnesses, perceived general health today, self-perceived general health in the past 6 months | PHC utilization in the past 6 months | (r = 0.056)-NS | NA-Excluded in the model |
|  |  |  | Age, education, employment status, tobacco use, chronic illnesses, perceived general health today, self-perceived general health in the past 12 months | PHC utilization in the past 12 months | (r = 0.002)-NS | NA-Excluded in the model |
| Health Insurance coverage (insured/uninsured) | Albanese et al., 2011 | Poisson Regression | Age, gender, educational level, number of assets, currently married, living with children, ICD-10 depression, dementia, number of physical illnesses | Use of any community health care services in the past three months | NA | Dominican Republic (aPR = 1.19, 95%CI 1.07-1.32); Puerto Rico (aPR = 1.42, 95%CI 1.19-1.71); Venezuela (aPR = 1.11, 95%CI 1.04-1.19); Peru urban (aPR = 1.69, 95%CI 1.39-2.05); Peru rural (aPR = 1.20, 95%CI 0.87-1.65); Mexico urban (aPR = 1.19, 95%CI 1.10-1.29); Mexico rural (aPR = 1.22, 95%CI 1.12-1.34); China urban (aPR = 2.02, 95%CI 1.36-3.00); China rural (aPR = 1.63, 95%CI 0.84-3.16); India urban (aPR = 1.02, 95%CI 0.70-1.50); India rural- Too few exposed; Nigeria- Too few exposed; Meta-analytical pooled effect (aPR = 1.27, 95%CI 1.16-1.38) |
| Health insurance coverage (insured/uninsured) | Goeppel et al., 2016 | Logistic Regression | Sex, age, place of residence, educational level, income quintile, comorbidity | Access to basic chronic care |  | Ref. uninsured, China (OR = 1.54, 95%CI 1.02-2.33); Ghana (OR = 1.69, 95%CI 1.25-2.28); India (OR = 3.03, 95%CI 1.88-4.87); Mexico OR = 2.73, 95%CI 1.40- 5.33); South Africa (OR = 1.01, 95%CI 0.67-1.52)  Russian Federation: N/A |
| Health insurance coverage (insured/uninsured) | He et al., 2013 | Logistic Regression | Age, gender, educational level, chronic diseases, walking time to community health service center, medical expenses in the latest year | Use of community health services during the past year | Health insurance (χ^2^ = 98.309, p<0.001) | Ref. No health insurance, Having health insurance (OR = 59.213, 95%CI 31.947-109.749) |
| Health insurance types (self-fund/basic medical insurance for urban workers/free medical treatment/new rural cooperative medical insurance/others) | Sun et al., 2013 | Chi-Square Test & Logistic Regression | Urban and rural areas, age, marital status, level of education, occupation, household income, living status, chronic disease conditions, self-care dependency | Use of community health services in the past year | χ^2^ = 106.157, P<0.001 | Ref. receiving any types of health insurance, self-fund (OR = 0.128, 95%CI 0.099-0.166) |
| Health insurance (reimbursed/unreimbursed) | Lu et al., 2015 | Logistic Regression | Gender, education, living status, monthly income, chronic diseases, willingness of seeking treatments in community when illness | Use of community health services | NA | (1) Chronic disease management services: Ref. unreimbursed insurance, reimbursed (OR = 2.83, 95%CI 1.29-6.19);  (2) Health records management services: Ref. unreimbursed insurance, reimbursed (OR = 1.86, 95%CI 1.12-3.10);  (3) Family visit service: Ref. unreimbursed insurance, reimbursed (OR = 1.76, 95%CI 1.11-2.79);  (4) Health checkup services: Ref. unreimbursed insurance, reimbursed (OR = 2.97, 95%CI 1.92-4.58);  (5) Dietary guidance services: Ref. unreimbursed insurance, reimbursed (OR = 1.82, 95%CI 1.01-3.26);  (6) Health education and counselling services: Ref. unreimbursed insurance, reimbursed (OR = 2.18, 95%CI 1.10-4.31) |
| Health insurance types (self-fund/urban basic health insurance/free medical treatment/other social health insurance) | Xi et al., 2010 | Logistic Regression | Age, gender, education, monthly income (poverty), occupation (before 60 years old), living status, chronic disease, severity of disease, distance to community care center, awareness of community health service | Use of community health services during the past year | NA | NS- Excluded in the model |

** NA: Not available*

*NS: Not significant*

**S1 Appendix*.* PRISMA 2009 Checklist**

| **Section/topic** | **#** | **Checklist item** | **Reported on page #** |
| --- | --- | --- | --- |
| **TITLE** | | |  |
| Title | 1 | Identify the report as a systematic review, meta-analysis, or both. | 1 |
| **ABSTRACT** | | |  |
| Structured summary | 2 | Provide a structured summary including, as applicable: background; objectives; data sources; study eligibility criteria, participants, and interventions; study appraisal and synthesis methods; results; limitations; conclusions and implications of key findings; systematic review registration number. | 1 |
| **INTRODUCTION** | | |  |
| Rationale | 3 | Describe the rationale for the review in the context of what is already known. | 1-2 |
| Objectives | 4 | Provide an explicit statement of questions being addressed with reference to participants, interventions, comparisons, outcomes, and study design (PICOS). | 2-3 |
| **METHODS** | | |  |
| Protocol and registration | 5 | Indicate if a review protocol exists, if and where it can be accessed (e.g., Web address), and, if available, provide registration information including registration number. | 3 |
| Eligibility criteria | 6 | Specify study characteristics (e.g., PICOS, length of follow-up) and report characteristics (e.g., years considered, language, publication status) used as criteria for eligibility, giving rationale. | 3-4 |
| Information sources | 7 | Describe all information sources (e.g., databases with dates of coverage, contact with study authors to identify additional studies) in the search and date past searched. | 4-5 |
| Search | 8 | Present full electronic search strategy for at least one database, including any limits used, such that it could be repeated. | 3 |
| Study selection | 9 | State the process for selecting studies (i.e., screening, eligibility, included in systematic review, and, if applicable, included in the meta-analysis). | 4-5 |
| Data collection process | 10 | Describe method of data extraction from reports (e.g., piloted forms, independently, in duplicate) and any processes for obtaining and confirming data from investigators. | 5 |
| Data items | 11 | List and define all variables for which data were sought (e.g., PICOS, funding sources) and any assumptions and simplifications made. | 5 |
| Risk of bias in individual studies | 12 | Describe methods used for assessing risk of bias of individual studies (including specification of whether this was done at the study or outcome level), and how this information is to be used in any data synthesis. | 5 |
| Summary measures | 13 | State the principal summary measures (e.g., risk ratio, difference in means). | 6 |
| Synthesis of results | 14 | Describe the methods of handling data and combining results of studies, if done, including measures of consistency (e.g., I^2^) for each meta-analysis. | 6 |

Page 1 of 2

| **Section/topic** | **#** | **Checklist item** | **Reported on page #** |
| --- | --- | --- | --- |
| Risk of bias across studies | 15 | Specify any assessment of risk of bias that may affect the cumulative evidence (e.g., publication bias, selective reporting within studies). |  |
| Additional analyses | 16 | Describe methods of additional analyses (e.g., sensitivity or subgroup analyses, meta-regression), if done, indicating which were pre-specified. |  |
| **RESULTS** | | |  |
| Study selection | 17 | Give numbers of studies screened, assessed for eligibility, and included in the review, with reasons for exclusions at each stage, ideally with a flow diagram. | 6-7 |
| Study characteristics | 18 | For each study, present characteristics for which data were extracted (e.g., study size, PICOS, follow-up period) and provide the citations. | 6-7 |
| Risk of bias within studies | 19 | Present data on risk of bias of each study and, if available, any outcome level assessment (see item 12). | 6 |
| Results of individual studies | 20 | For all outcomes considered (benefits or harms), present, for each study: (a) simple summary data for each intervention group (b) effect estimates and confidence intervals, ideally with a forest plot. | 7 |
| Synthesis of results | 21 | Present results of each meta-analysis done, including confidence intervals and measures of consistency. | 7-11 |
| Risk of bias across studies | 22 | Present results of any assessment of risk of bias across studies (see Item 15). |  |
| Additional analysis | 23 | Give results of additional analyses, if done (e.g., sensitivity or subgroup analyses, meta-regression [see Item 16]). |  |
| **DISCUSSION** | | |  |
| Summary of evidence | 24 | Summarize the main findings including the strength of evidence for each main outcome; consider their relevance to key groups (e.g., health care providers, users, and policy makers). | 11-15 |
| Limitations | 25 | Discuss limitations at study and outcome level (e.g., risk of bias), and at review-level (e.g., incomplete retrieval of identified research, reporting bias). | 13-14 |
| Conclusions | 26 | Provide a general interpretation of the results in the context of other evidence, and implications for future research. | 15-16 |
| **FUNDING** | | |  |
| Funding | 27 | Describe sources of funding for the systematic review and other support (e.g., supply of data); role of funders for the systematic review. |  |

*From:* Moher D, Liberati A, Tetzlaff J, Altman DG, The PRISMA Group (2009). Preferred Reporting Items for Systematic Reviews and Meta-Analyses: The PRISMA Statement. PLoS Med 6(7): e1000097. doi:10.1371/journal. pmed1000097

For more information, visit: **www.prisma-statement.org**.

Page 2 of 2

**S2 Appendix*.* Full Searching Strategy**

**Database: Embase, Medline, Psych Info, Global Health**

1. (Afghanistan or Albania or Algeria or "American Samoa" or Angola or "Antigua and Barbuda" or Argentina or Armenia or Aruba or Azerbaijan or Bahrain or Barbados or Bangladesh or Belarus or Belize or Benin or Bhutan or Bolivia or "Bosnia and Herzegovina" or Botswana or Brazil or Bulgaria or "Burkina Faso" or Burundi or Cambodia or "Cabo Verde" or Cameroon or "Central African Republic" or Chad or Chile or China or Colombia or Comoros or "Congo Republic" or "Costa Rica" or "C?te d'Ivoire" or "Ivory Coast" or Czechoslovakia or Croatia or Cuba or "Czech Republic" or Djibouti or Dominica or "Dominican Republic" or Ecuador or "El Salvador" or "Egypt, Arab Republic" or "Equatorial Guinea" or Eritrea or Estonia or Ethiopia or Fiji or Gabon or Gambia or Georgia or Ghana or Grenada or Guatemala or Guinea or "Guinea-Bissau" or Guyana or Gibraltar or Greece or Guam or Haiti or Hungary or Honduras or India or Indonesia or Iran or Iraq or "Isle of Man" or Jamaica or Jordan or Kazakhstan or Kenya or Kiribati or Kosovo or "Korea, Dem? Republic" or "North Korea" or "South Korea" or "Korea, Republic" or Kenya or "Kyrgyz Republic" or "Lao PDR" or Latvia or Lebanon or Libya or Liberia or Lithuania or Lesotho or "Macao SAR, China" or "Macedonia, FYR" or Madagascar or Malaysia or Malawi or Maldives or Mali or Malta or "Marshall Islands" or Mauritania or Mauritius or Mexico or Mayotte or Micronesia or Moldova or Mongolia or Montenegro or Morocco or Mozambique or Macedonia or Myanmar or Namibia or "New Caledonia" or "Netherlands Antilles" or "Northern Mariana Islands" or Nicaragua or Nepal or Nauru or Niger or Nigeria or Oman or Pakistan or Palau or Panama or "Papua New Guinea" or Paraguay or Peru or Philippines or Poland or Portugal or "Puerto Rico" or Romania or "Russian Federation" or Rwanda or Samoa or "Saudi Arabia" or "S?o Tom? and Principe" or Senegal or Serbia or "Serbia and Montenegro" or Seychelles or "Sierra Leone" or "Slovak Republic" or Slovenia or "Solomon Islands" or "South Africa" or Somalia or "Sri Lanka" or "Saint Kitts and Nevis" or "St? Kitts and Nevis" or "Saint Lucia" or "St? Lucia" or "Saint Vincent and the Grenadines" or "St? Vincent and the Grenadines" or South Sudan or Sudan or Suriname or Swaziland or "Syrian Arab Republic" or Tajikistan or Tanzania or Thailand or Togo or Tonga or "Trinidad and Tobago" or Tunisia or Turkey or "Timor-Leste" or Turkmenistan or Tuvalu or Uganda or Ukraine or Uruguay or USSR or Uzbekistan or Vanuatu or "Venezuela, RB" or "Bolivarian Republic of Venezuela" or Vietnam or "West Bank and Gaza" or Yemen or Yugoslavia or Zimbabwe or Zambia).hw,kf,ti,ab,cp.

2. (Africa or Asia or Caribbean or West Indies or South America or Latin America or Central America).hw,kf,ti,ab,cp.

3. Developing Countries.sh,kf.

4. ("Low and Middle income countr*" or "Low* Middle-income countr*" or "lmic*" or

"lami countr*").ti,ab.

5. ((developing or less* developed or under developed or underdeveloped or low* middle income or low* income or underserved or deprived or poor*) adj (countr* or nation? or state? or population? or world or econom*)).ti,ab.

6. ((developing or less* developed or under developed or underdeveloped or low* middle income or low* income) adj (economy or economies)).ti,ab.

7. (low* adj (gdp or gnp or gross domestic or gross national)).ti,ab.

8. (Transition* adj (countr* or econom*)).ti,ab.

9. 1 or 2 or 3 or 4 or 5 or 6 or 7 or 8

10. ("older people" or "older adult*" or "older population" or "older group" or "old

age" or "old*" or "elder*" or "aged" or "ageing" or "aging").mp.

11. ("gender*" or "sex" or "men" or "women" or "man" or "woman" or "male" or "female").ti,ab.

12. exp "socioeconomic status"/

13. exp "socioeconomic factor?"/

14. exp "social class"/

15. exp income/

16. exp education/

17. exp poverty/

18. exp inequality/

19. exp deprivation/

20. ("socioeconomic position" or "socioeconomic level").mp.

21. "assets index".ti,ab.

22. occupation.ti,ab.

23. pension.ti,ab.

24. socioeconomics/or economic aspect/

25. (social class status or level?).ti,ab.

26. (education status or level?).ti,ab.

27. salary/income or earning?.mp. or wage?.mp. or poor.mp. or wealth.mp.

28. (unemploy* or (employment adj2 (status or indicator? or level?))).ti,ab.

29. ((socioeconomic or socio-economic or economic) adj2 (factor? or inequalit* or

indicator? or status or development)).ti,ab.

30. ((household? or famil*) adj3 (income or earning? or wage? or poor or wealth)).ti,ab.

31. (insurance status or indicator? or level?).ti,ab.

32. 11 or 12 or 13 or 14 or 15 or 16 or 17 or 18 or 19 or 20 or 21 or 22 or 23 or 24 or 25 or 26 or 27 or 28 or 29 or 30 or 31

33. 12 or 13 or 14 or 15 or 16 or 17 or 18 or 19 or 20 or 21 or 22 or 23 or 24 or 25 or 26 or 27 or 28 or 29 or 30 or 31

34. exp "primary healthcare"/or "primary healthcare".mp.

35. exp "primary healthcare"/or "primary healthcare".mp.

36. "primary health service".mp.

37. exp "primary care"/or "primary care".mp.

38. "traditional heal*".ti,ab.

39. "faith heal*".ti,ab.

40. "religious heal*".ti,ab.

41. "spiritual heal*".ti,ab.

42. "traditional medicine".ti,ab.

43. "community care".ti,ab.

44. "community healthcare".ti,ab.

45. "community healthcare".ti,ab.

46. "community rehabilitation".ti,ab.

47. "community health service*".ti,ab.

48. "basic public health service*".ti,ab.

49. "population-based intervention".ti,ab.

50. "health center".ti,ab.

51. "health centre".ti,ab.

52. "health station".ti,ab.

53. "first level hospital".ti,ab.

54. "township hospital".ti,ab.

55. "village clinic".ti,ab.

56. "GP".ti,ab.

57. "general practic*".ti,ab.

58. 34 or 35 or 36 or 37 or 38 or 39 or 40 or 41 or 42 or 43 or 44 or 45 or 46 or 47 or 48 or 49 or 50 or 51 or 52 or 53 or 54 or 55 or 56 or 57

59. (Access* or visit* or use* or using* or usage or utili?ation).ti,ab.

60. 9 and 10 and 32 and 58 and 59

**CNKI Database**

((TI=‘基层卫生’) OR (TI=‘基层医疗’) OR (TI=‘初级卫生’) OR (TI=‘社区医疗’) OR (TI=‘社区卫生’) OR (TI=‘乡村医疗’) OR (TI=‘乡村卫生’) OR (TI=‘基本公共卫生’) OR (TI=‘中医’) OR (AB=‘基层卫生’) OR (AB=‘基层医疗’) OR (AB=‘初级卫生’) OR (AB=‘社区医疗’) OR (AB=‘社区卫生’) OR (AB=‘乡村医疗’) OR (AB=‘乡村卫生’) OR (AB=‘基本公共卫生’) OR (AB=‘中医’)) AND ((TI=‘服务利用’) OR (TI=‘服务使用’) OR AB=‘服务利用’) OR (AB=‘服务使用’)) AND ((FT=‘老年人’) OR (FT=‘老人’) OR (FT=‘老龄’)) AND ((TI=‘社会经济地位’) OR (TI=‘社会经济指标’) OR (TI=‘社会阶层’) OR (TI=‘资产指数’) OR (TI=‘收入’) OR (TI=‘教育’) OR (TI=‘贫穷’) OR (TI=‘就业’) OR (TI=‘职业’) OR (TI=‘家庭收入’) OR (TI=‘保险’) OR (TI=‘性别’) OR (TI=‘男’) OR (TI=‘女’) OR (AB=‘社会经济地位’) OR (AB=‘社会经济指标’) OR (AB=‘社会阶层’) OR (AB=‘资产指数’) OR (AB=‘收入’) OR (AB=‘教育’) OR (AB=‘贫穷’) OR (AB=‘就业’) OR (AB=‘职业’) OR (AB=‘家庭收入’) OR (AB=‘保险’) OR (AB=‘性别’) OR (AB=‘男’) OR (AB=‘女’))

**LILACS Database**

(ab:(("older people" OR "older adult" OR "older population" OR "older group" OR "old age" OR "elder" OR "aged" OR "ageing" OR "aging"))) AND (ab:(("socioeconomic status" OR "socioeconomic factor?" OR "social class" OR income OR education OR poverty OR inequality OR deprivation OR "socioeconomic position" OR "socioeconomic level" OR "assets index" OR occupation OR pension OR "economic aspect" OR "social class" OR "social status" OR "social level" OR "education status" OR "education level" OR salary OR income OR earning OR wage OR poor OR wealth OR employment OR occupation OR "household income" OR insurance OR "gender" OR "sex" OR "men" OR "women" OR "man" OR "woman" OR "male" OR "female"))) AND (ab:(("primary healthcare" OR "primary healthcare" OR "primary healthcare" OR "primary healthcare" OR "primary health service" OR "primary care" OR "primary care" OR "traditional healing" OR "faith healing" OR "religious healing" OR "spiritual healing" OR "traditional medicine" OR "community care" OR "community healthcare" OR "community healthcare" OR "community rehabilitation" OR "community health service" OR "basic public health service" OR "population-based intervention" OR "health center" OR "health centre" OR "health station" OR "first level hospital" OR "township hospital" OR "village clinic" OR "GP" OR "general practice"))) AND (ab:((access OR visit OR use OR using OR usage OR utilization OR utilization))) AND (instance:"regional") AND ( db:("LILACS"))
